# Joint registration and synthesis using a probabilistic model for alignment of MRI and histological sections

**DOI:** 10.1016/j.media.2018.09.002

**Published:** 2018-09-22

**Authors:** Juan Eugenio Iglesias, Marc Modat, Loïc Peter, Allison Stevens, Roberto Annunziata, Tom Vercauteren, Ed Lein, Bruce Fischl, Sebastien Ourselin

**Affiliations:** a Translational Imaging Group, Centre for Medical Image Computing, University College London, UK; b Wellcome EPSRC Centre for Interventional and Surgical Sciences (WEISS), University College London, UK; c Martinos Center for Biomedical Imaging, Harvard Medical School and Massachusetts General Hospital, USA; d Allen Institute for Brain Science, USA; e Computer Science and AI lab, Massachusetts Institute of Technology, USA

**Keywords:** Synthesis, Registration, Variational bayes, Histology reconstruction

## Abstract

Nonlinear registration of 2D histological sections with corresponding slices of MRI data is a critical step of 3D histology reconstruction algorithms. This registration is difficult due to the large differences in image contrast and resolution, as well as the complex nonrigid deformations and artefacts produced when sectioning the sample and mounting it on the glass slide. It has been shown in brain MRI registration that better spatial alignment across modalities can be obtained by synthesising one modality from the other and then using intra-modality registration metrics, rather than by using information theory based metrics to solve the problem directly. However, such an approach typically requires a database of aligned images from the two modalities, which is very difficult to obtain for histology and MRI.

Here, we overcome this limitation with a probabilistic method that simultaneously solves for deformable registration and synthesis directly on the target images, without requiring any training data. The method is based on a probabilistic model in which the MRI slice is assumed to be a contrast-warped, spatially deformed version of the histological section. We use approximate Bayesian inference to iteratively refine the probabilistic estimate of the synthesis and the registration, while accounting for each other’s uncertainty. Moreover, manually placed landmarks can be seamlessly integrated in the framework for increased performance and robustness.

Experiments on a synthetic dataset of MRI slices show that, compared with mutual information based registration, the proposed method makes it possible to use a much more flexible deformation model in the registration to improve its accuracy, without compromising robustness. Moreover, our framework also exploits information in manually placed landmarks more efficiently than mutual information: landmarks constrain the deformation field in both methods, but in our algorithm, it also has a positive effect on the synthesis – which further improves the registration. We also show results on two real, publicly available datasets: the Allen and BigBrain atlases. In both of them, the proposed method provides a clear improvement over mutual information based registration, both qualitatively (visual inspection) and quantitatively (registration error measured with pairs of manually annotated landmarks).

## 1 Introduction

### 1.1 Motivation: human brain atlases

Histology is the study of tissue microanatomy. Histological analysis involves cutting a wax-embedded or frozen block of tissue into very thin sections (in the order of 10 microns), which are subsequently stained, mounted on glass slides, and examined under the microscope. Using different types of stains, different microscopic structures can be enhanced and studied. Moreover, mounted sections can be digitised at high resolution – in the order of a micron. Digital histological sections not only enable digital pathology in a clinical setting, but also open the door to an array of image analysis applications.

A promising application of digital histology is the construction of high resolution computational atlases of the human brain. Such atlases have traditionally been built using MRI scans and/or associated manual segmentations, depending on whether they describe image intensities, neuroanatomical label probabilities, or both. Examples include: the MNI atlas (Evans et al., 1993; Collins et al., 1994), the Colin 27 atlas (Holmes et al., 1998), the ICBM atlas (Mazziotta et al., 1995; 2001), and the LONI LPBA40 atlas (Shattuck et al., 2008).

Computational atlas building using MRI is limited by the resolution and contrast that can be achieved with this imaging technique. The resolution barrier can be partly overcome with *ex vivo* MRI, in which motion – and hence time constraints – are eliminated, enabling longer acquisition at ultra-high resolution (~100 μm), which in turns enables manual segmentation at a higher level of detail (Augustinack et al., 2005; Yushkevich et al., 2009; Iglesias et al., 2015; Saygin et al., 2017). However, not even the highest resolution achievable with *ex vivo* MRI is sufficient to study microanatomy. Moreover, and despite recent advances in pulse sequences, MRI does not generate visible contrast at the boundaries of many neighbouring brain structures, the way that histological staining does.

For these reasons, recent studies building computational brain atlases are using stacks of digitised histological sections, which enable more accurate manual segmentations, to build atlases at a superior level of detail. Examples include the work by Chakravarty et al. (2006) on the thalamus and basal ganglia; by Krauth et al. (2010) on the thalamus; by Adler et al. (2014, 2016, 2018) on the hippocampus; our recent work on the thalamus (Iglesias et al., 2017), and the recently published atlas from the Allen Institute (Ding et al., 2016).^3^

### 1.2 Related work on 3D histology reconstruction

The main drawback of building atlases with histology is the fact that the 3D structure of the tissue is lost in the processing. Sectioning and mounting introduce large nonlinear distortions in the tissue structure, including artefacts such as folds and tears. In order to recover the 3D shape, image registration algorithms can be used to estimate the spatial correspondences between the different sections. This problem is commonly known as “histology reconstruction” (Pichat et al., 2018).

The simplest approach to histology reconstruction is to sequentially align sections in the stack to their neighbours using a linear registration method. There is a wide literature on the topic, not only for histological sections but also for autoradiographs. Most of these methods use robust registration algorithms, e.g., based on edges (Hibbard and Hawkins, 1988; Rangarajan et al., 1997), block matching (Ourselin et al., 2001) or point disparity (Zhao et al., 1993). There are also nonlinear versions of serial registration methods (e.g., Arganda-Carreras et al. 2010; Pitiot et al. 2006; Chakravarty et al. 2006; Schmitt et al. 2007), some of which introduce smoothness constraints to minimise the impact of sections that are heavily affected by artefacts and/or are poorly registered (Ju et al., 2006; Yushkevich et al., 2006; Cifor et al., 2011; Iglesias et al., 2018).

The problem with serial alignment of sections is that, without any information on the original shape, methods are prone to accumulating errors along sections (known as *“z-shift”*) and to straightening curved structures (known as *“banana effect”*, since the reconstruction of a sliced banana would be a cylinder). One way of overcoming this problem is the use of fiducial markers such as needles or rods (e.g., Humm et al. 2003); however, this approach has two disadvantages: the tissue may be damaged by the needles, and additional bias can be introduced in the registration if the sectioning plane is not perpendicular to the needles.

Another way of combating the *“z-shift”* and *banana effect* is to use an external reference volume without geometric distortion. In an early study, Kim et al. (1997) used video frames to construct such reference, in the context of autoradiograph alignment. More recent works have used MRI scans (e.g., Malandain et al. 2004; Dauguet et al. 2007; Yang et al. 2012; Ebner et al. 2017). The general idea is to iteratively update: 1. a rigid transform bringing the MRI to the space of the histological stack; and 2. a nonlinear transform per histological section, which registers it to the space of the corresponding (resampled) MRI plane. A potential advantage of using MRI as a reference frame for histology reconstruction is that one recovers in MRI space the manual delineations made on the histological sections, which can be desirable when building atlases (Adler et al., 2016; 2018).

Increased stability in histology reconstruction can be obtained by using a third, intermediate modality to assist the process. Such modality is typically a stack of blockface photographs, which are taken prior to sectioning and are thus spatially undistorted. Such photographs help bridge the spaces of the MRI (neither modality is distorted) and the histology (plane correspondences are known). An example of this approach is the BigBrain project (Amunts et al., 2013).

Assuming that a good estimate of the rigid alignment between the MRI and the histological stack is available, the main technical challenge of 3D histology reconstruction is the nonlinear 2D registration of a histological section with the corresponding (resampled) MRI plane. These images exhibit very different contrast properties, in addition to modality-specific artefacts, e.g., tears in histology, bias field in MRI. Therefore, generic information theory based registration metrics such as mutual information (Maes et al., 1997; Wells et al., 1996; Pluim et al., 2003) yield unsatisfactory results. This is partly due to the fact that such approaches only capture statistical relationships between image intensities at the voxel level, disregarding geometric information.

### 1.3 Related work on image synthesis for registration

An alternative to mutual information for inter-modality registration is to use image synthesis. The premise is simple: if we need to register a floating image *F_A_* of modality *A* to a reference image *R_B_* of modality *B*, and we have access to a dataset of spatially aligned pairs of images of the two modalities {*A_i_*, *B_i_*}, then we can: estimate a synthetic version of the floating image *F_B_* that resembles modality *B*; register *F_B_* to *R_B_* with an intra-modality registration algorithm; and apply the resulting deformation field to the original floating image *F_A_*. In the context of brain MRI, we have shown in Iglesias et al. (2013) that such an approach, even with a simple synthesis model (Hertzmann et al., 2001), clearly out-performs registration based on mutual information. This result has been replicated in other studies (e.g., Roy et al., 2014), and similar conclusions have been reached in the context of MRI segmentation (Roy et al., 2013) and classification (van Tulder and de Bruijne, 2015).

Medical image synthesis has gained popularity in the last few years due to the advent of hybrid PET-MR scanners, since synthesising a realistic CT scan from the corresponding MR enables accurate attenuation correction of the PET data (Burgos et al., 2014; Huynh et al., 2016). Another popular application of CT synthesis from MRI is dose calculation in radiation therapy (Kim et al., 2015; Siversson et al., 2015). Unfortunately, most of these synthesis algorithms are based on supervised machine learning techniques, which require aligned pairs of images from the two modalities – which are very hard to obtain for histology and MRI.

A possible alternative to supervised synthesis is a weakly supervised paradigm, best represented by the recent deep learning method CycleGAN (Zhu et al., 2017). This algorithm uses two sets of (unpaired) images of the two modalities, to learn two mapping functions, from each modality to the other. CycleGAN enforces cycle consistency of the two mappings (i.e., that they approximately invert each other), while training two classifiers that discriminate between synthetic and real images of each modality in order to avoid overfitting. While this technique has been shown to produce realistic medical images (Chartsias et al., 2017; Wolterink et al., 2017), it has an important limitation in the context of histology-MRI registration: it is unable to exploit the pairing between the (nonlinearly misaligned) histology and MRI images. Another disadvantage of CycleGAN is that, since a database of cases is necessary to train the model, it cannot be applied to a single image pair, i.e., it cannot be used as a generic inter-modality registration tool.

### 1.4 Contribution

In this study, we propose a novel probabilistic model that *simultaneously* solves for registration and synthesis directly on the target images, i.e., without any training data. The principle behind the method is that improved registration provides less noisy data for the synthesis, while more accurate synthesis leads to better registration. Our framework enables these two components to iteratively exploit the improvements in the estimates of the other, while considering the uncertainty in each other’s parameters. Taking uncertainty into account is crucial: if one simply tries to iteratively optimise synthesis and registration while keeping the other fixed to a point estimate, both components are greatly affected by the noise introduced by the other. More specifically, misregistration leads to bad synthesis due to noisy training data, whereas accurate registration to a poorly synthesised image yields incorrect alignment.

If multiple image pairs are available, the framework exploits the complete database, by jointly considering the probabilistic registrations between the pairs. In addition, the synthesis algorithm effectively takes advantage of the spatial structure in the data, as opposed to mutual information based registration. Moreover, the probabilistic nature of the model also enables the seamless integration of manually placed landmarks, which inform both the registration (directly) and the synthesis (indirectly, by creating areas of high certainty in the registration); the results show that the improvement in synthesis yields more accurate registration than when the landmarks only inform the deformation field. Finally, we present a variational expectation maximisation algorithm (VEM, also known as variational Bayes) to solve the model with Bayesian inference, and illustrate the proposed approach through experiments on synthetic and real data.

The rest of this paper is organised as follows. In Section 2, we describe the probabilistic model on which our algorithm relies (Section 2.1), as well as an inference algorithm to compute the most likely solution within the proposed framework (Section 2.2). In Section 3, we describe the MRI and histological data (Section 3.1) that we used in our experiments (Section 3.2), as well as the results on real data and the Allen atlas (Section 3.3). Finally, Section 4 concludes the paper.

## 2 Methods

### 2.1 Probabilistic framework

**Fig. 1:**
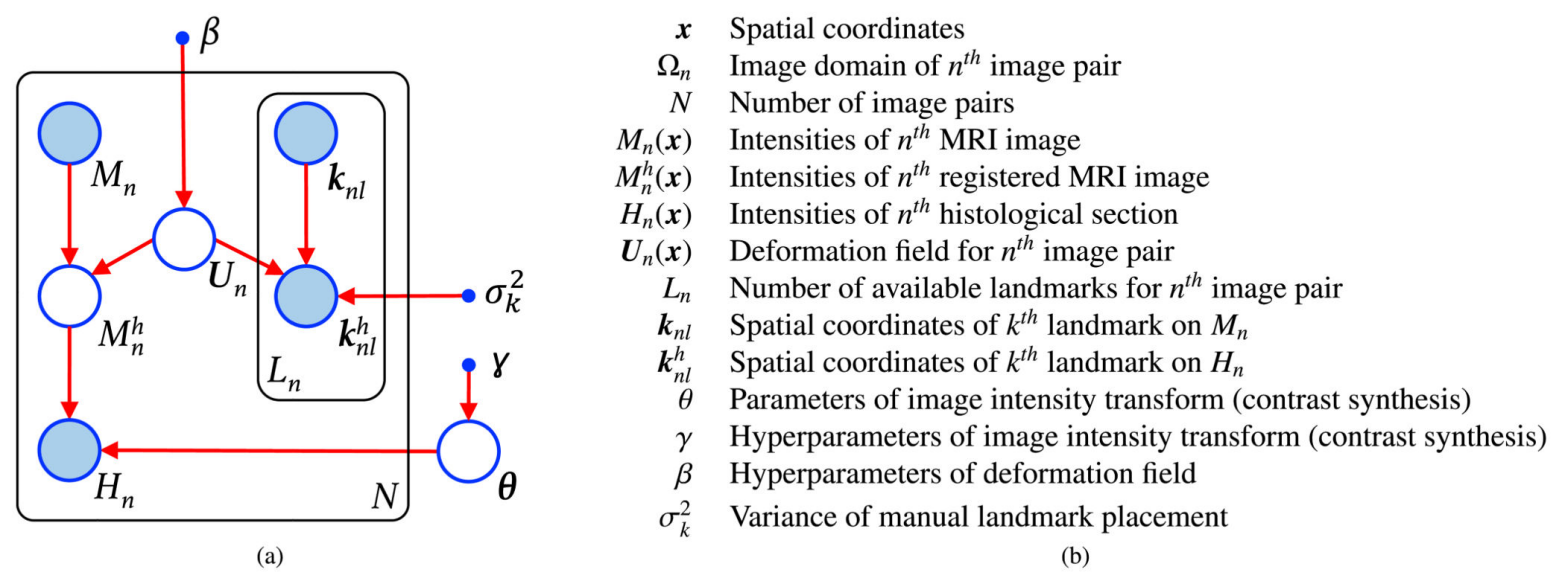
(a) Graphical model of the proposed probabilistic framework. Circles represent random variables or parameters, arrows indicate dependencies between the variables, dots represent known (hyper)parameters, shaded variables are observed, and plates indicate replication. (b) Mathematical symbols corresponding to the model.

The graphical model of our probabilistic framework and corresponding mathematical symbols are shown in Fig. 1. For the sake of simplicity, we describe the framework from the perspective of the MRI to histology registration problem, though the method is general and can be applied to other inter-modality registration task – in any number of dimensions.

Let {*M_n_*}_*n*=1,…,*N*_ and {*H_n_*}_*n*=1,…,*N*_ represent *N* ≥ 1 MRI image slices and corresponding histological sections. We assume that each pair of images has been coarsely aligned with a 2D linear registration algorithm (e.g., using mutual information), and are hence defined over the same image domain Ω_*n*_. *M_n_* and *H_n_* are functions of the spatial coordinates ***x*** ∈ Ω_*n*_, i.e., *M_n_* = *M_n_* (***x***) and *H_n_* = *H_n_* (***x***). In addition, let *K_n_* and $K_{n}^{h}$ represent two sets of *L_n_* corresponding landmarks, manually placed on the *n^th^* MRI image and histological section, respectively: *K_n_* = {***k**_nl_*}_*l*=1,…,*L_n_*_ and $K_{n}^{h}={\left\{{\text{k}}_{nl}^{h}\right\}}_{l=1,\ldots ,L_{n}},$ where ***k**_nl_* and ${\text{k}}_{nl}^{h}$ are 2D vectors with the spatial coordinates of the *l^th^* landmark on the *n^th^* image pair; for reasons that will be apparent in Section 2.2 below, we will assume that every ***k**_nl_* coincides with an integer pixel coordinate. Finally, $M_{n}^{h}$ represents the *n^th^* MR image after applying a nonlinear deformation field ***U**_n_*(***x***), which deterministically warps it to the space of the *n^th^* histological section *H_n_*, i.e.,

(1) $$M_{n}^{h}\left(\text{x}\right)=M_{n}\left(\text{x}+{\text{U}}_{n}\left(\text{x}\right)\right),$$

which in general requires interpolation of *M_n_*(***x***).

Each deformation field ***U**_n_* is assumed to be an independent sample of a Markov Random Field (MRF) prior, with unary potentials penalising large displacements (their squared module), and binary potentials penalising the squared gradient magnitude:

(2) $$p\left({\text{U}}_{n}\right)=\frac{1}{Z_{n}\left(\beta_{1},\beta_{2}\right)}\prod_{\text{x}\in \Omega_{n}}e^{-\beta_{1}∥{\text{U}}_{n}\left(\text{x}\right){∥}^{2}-\beta_{2}\sum_{{\text{x}}^{\prime }\in Β\left(\text{x}\right)}∥{\text{U}}_{n}\left(\text{x}\right)-{\text{U}}_{n}\left({\text{x}}^{\prime }\right){∥}^{2}},$$

where *β*_1_ > 0 and *β*_2_ > 0 are the parameters of the MRF (which we group in *β* = {*β*_1_, *β*_2_}); *Z_n_*(*β*_1_, *β*_2_) is the partition function; and *ℬ*(***x***) is the neighbourhood of the pixel located at ***x***. We note that this prior encodes a regularisation similar to that of the popular demons registration algorithm (Vercauteren et al., 2007; Cachier et al., 2003). Moreover, we also discretise the deformation fields, such that ***U**_n_*(***x***) can only take values in a finite, discrete set of displacements {**Δ**_*s*_}_*s*=1,…,*S*_ at any location, i.e., ***U**_n_*(***x***) ∈ {**Δ**_*s*_}. We note that these displacements do not need to be integer (in pixels). While this choice of deformation model and regulariser does not guarantee the registration to be diffeomorphic (which might be desirable), it enables marginalisation over the deformation fields {***U**_n_*} – and, as we will discuss in Section 2.2 below, a more sophisticated deformation model can be used to refine the final registration.

Application of ***U**_n_* to *M_n_* and *K_n_* yields not only a registered MRI image $M_{n}^{h}$ (Eq. (1)), but also a set of warped landmarks *K^h^*. When modelling *K^h^*, we need to account for the error made by the user when manually placing corresponding key-points in the MR images and the histological sections. We assume that these errors are independent and follow zero-mean, isotropic Gaussian distributions parametrised by their covariances $\sigma_{k}^{2}\text{I}$ (where ***I*** is the 2 × 2 identity matrix, and where $\sigma_{k}^{2}$ is expected to be quite small):

(3) $$\begin{array}{l} p\left(K_{n}^{h}|K_{n},{\text{U}}_{n},\sigma_{k}^{2}\right)=\prod_{l=1}^{L_{n}}p\left({\text{k}}_{nl}^{h}|{\text{k}}_{nl}-{\text{U}}_{n}\left({\text{k}}_{nl}^{h}\right),\sigma_{k}^{2}\right)\\ \;=\prod_{l=1}^{L_{n}}\frac{1}{2\pi \sigma_{k}^{2}}\text{exp}\left[-\frac{1}{2\sigma_{k}^{2}}∥{\text{k}}_{nl}^{h}-{\text{k}}_{nl}+{\text{U}}_{n}\left({\text{k}}_{nl}^{h}\right){∥}^{2}\right]. \end{array}$$

Note that the parameter $\sigma_{k}^{2}$ is assumed to have the same value for all landmark pairs. While we would expect the variance of the error to be larger in flat areas of the image (we could make it dependent on e.g., the gradient magnitude), we will here assume that the landmarks will seldom be located around such uniform areas – as the user would normally use salient features (e.g., corners) as reference points.

Finally, to model the connection between the intensities of the histological sections {*H_n_*} and the registered MRI images $\{M_{n}^{h}\},$ we follow Tu et al. (2008) and make the assumption that:

(4) $$p\left(H_{n}|M_{n}^{h},\theta \right)\;\propto \;p\left(M_{n}^{h}|H_{n},\theta \right).$$

This assumption is equivalent to adopting a discriminative approach to model the contrast synthesis. While this discriminative component breaks the generative nature of the framework, it also enables the modelling of much more complex relationships between the intensities of the two modalities, including spatial and geometric information about the pixels. Such spatial patterns cannot be captured by, e.g., mutual information, which only models statistical relationships between intensities (e.g., a random shuffling of pixels does not affect the metric). Any discriminative, probabilistic regression technique can be used to model the synthesis. Here we choose to use a regression forest (Breiman, 2001), which can model complex intensity relationships while being fast to train – which is crucial because we will have to retrain the forest several times in inference, as explained in Section 2.2 below. We assume conditional independence of the pixels in the prediction: the forest produces a Gaussian distribution for each pixel ***x*** separately, parametrised by *μ_n**x**_* and $\sigma_{n\text{x}}^{2}.$ Moreover, we place a (conjugate) Inverse Gamma prior on the variances $\sigma_{n\text{x}}^{2},$ with hyperparameters *a* and *b*:

(5) $$p\left(\sigma_{n\text{x}}^{2}|a,b\right)=\frac{b^{a}}{\Gamma \left(a\right)}{\left(\sigma_{n\text{x}}^{2}\right)}^{-a-1}\;\text{exp}\left(-b/\sigma_{n\text{x}}^{2}\right).$$

Thanks to the conjugacy property, this choice of prior greatly simplifies inference in Section 2.2 below, as it is equivalent to having observed 2*a* pseudo-samples (tree predictions) with sample variance *b*/*a*. The effect of the prior is to ensure that the Gaussians describing the predictions do not degenerate into zero variance distributions.

Henceforth, we use *θ* to represent the set of forest parameters, which groups the selected features, split values, tree structure and the prediction at each leaf node. The set of corresponding hyperparameters are grouped in *γ*, which includes the parameters of the Gamma prior {*a*, *b*}, the number of trees, and minimum number of samples in leaf nodes. The intensity model is hence:

$$\begin{array}{l} p\left(M_{n}^{h}|H_{n},\theta \right)=\prod_{\text{x}\in \Omega_{n}}p\left(M_{n}^{h}\left(\text{x}\right)|H_{n}\left(𝒲\left(\text{x}\right)\right),\theta \right)\\ \;=\prod_{\text{x}\in \Omega_{n}}𝒩(M_{n}^{h}\left(\text{x}\right);\mu_{n\text{x}}\left(H_{n}\left(𝒲\left(\text{x}\right)\right),\theta \right),\\ \;\sigma_{n\text{x}}^{2}\left(H_{n}\left(𝒲\left(\text{x}\right)\right),\theta \right)), \end{array}$$

where 𝒲(***x***) is a spatial window centred at ***x***, and *𝒩* represents the Gaussian distribution. Given the deterministic deformation model (Eq. (1)), and the assumption in Eq. (4), we finally obtain the likelihood term:

(6) $$\begin{array}{l} p\left(H_{n}|M_{n},{\text{U}}_{n},\theta \right)=\prod_{\text{x}\in \Omega_{n}}p\left(M_{n}\left(\text{x}+\text{U}\left(\text{x}\right)\right)|H_{n}\left(𝒲\left(\text{x}\right)\right),\theta \right)\\ \;=\prod_{\text{x}\in \Omega_{n}}𝒩(M_{n}\left(\text{x}+\text{U}\left(\text{x}\right)\right);\mu_{n\text{x}}\left(H_{n},\theta \right)\\ \;\sigma_{n\text{x}}^{2}\left(H_{n}\left(𝒲\left(\text{x}\right)\right),\theta \right)). \end{array}$$

We emphasise that, despite breaking the generative nature of the model, the assumption in Eq. (4) still leads to a valid objective function when performing Bayesian inference. This objective function can be optimised with standard inference techniques, as explained in Section 2.2 below.

### 2.2 Inference

We use Bayesian inference to “invert” the probabilistic model described in Section 2.1 above. If we group all the observed variables into the set $O=\left\{\left\{M_{n}\right\},\left\{H_{n}\right\},\left\{K_{n}\right\},\left\{K_{n}^{h}\right\},\beta ,\gamma ,\sigma_{k}^{2}\right\},$ the problem is to maximise:

(7) $$\begin{array}{l} \left\{{\hat{\text{U}}}_{n}\right\}=\underset{\left\{{\text{U}}_{n}\right\}}{\text{argmax}}\;p\left(\left\{{\text{U}}_{n}\right\}|O\right)=\underset{\left\{{\text{U}}_{n}\right\}}{\text{argmax}}\int_{\theta }p\left(\left\{{\text{U}}_{n}\right\}|\theta ,O\right)p\left(\theta |O\right)d\theta \\ \;\approx \underset{\left\{{\text{U}}_{n}\right\}}{\text{argmax}}\;p\left(\left\{{\text{U}}_{n}\right\}|\hat{\theta },O\right), \end{array}$$

where we have made the standard approximation that the posterior *p*(*θ*|*O*) is strongly peaked around its mode $\hat{\theta },$ i.e., we use point estimates for the parameters, computed as:

(8) $$\hat{\theta }=\underset{\theta }{\text{argmax}}\;p\left(\theta |O\right).$$

In this section, we first describe a VEM algorithm to obtain the point estimate of *θ* using Eq. (8) (Section 2.2.1), and then address the computation of the final registrations with Eq. (7) (Section 2.2.2). The presented method is summarised in Algorithm 1.

**Algorithm 1 Simultaneous synthesis and registration.**

**Input:** ${\left\{M_{n}\right\}}_{n=1,\ldots ,N},{\left\{H_{n}\right\}}_{n=1,\ldots ,N},K_{n},K_{n}^{h}$

**Output:** $\hat{\theta },\left\{{\hat{\text{U}}}_{n}\right\}$

     *q_n**x**_*(**Δ**) ← 1/*S*, ∀*n*, ***x***

     Initialise *θ* with Eq. (13) (random forest training)

     **while** $\mu_{n\text{x}},\sigma_{n\text{x}}^{2}$ changes **do**

          *E-step:*

          **for** *n* = 1 to *n* = *N* **do**

               Compute $\mu_{n\text{x}},\sigma_{n\text{x}}^{2}$ ∀***x*** ∈ Ω*n* with Eq. (14)

               **while** *q_n**x**_* changes **do**

                    Fixed point iteration of *q_n**x**_* (Eq. (12))

               **end while**

          **end for**

          *M-step:*

          Update *θ* with Eq. (13) (random forest retraining)

     **end while**

     $\hat{\theta }$ ← *θ*

     **for** *n* = 1 to *n* = *N* **do**

          Compute final $\mu_{n\text{x}},\sigma_{n\text{x}}^{2},\forall \text{x}\in \Omega_{n}$ with Eq. (14)

          Compute ${\hat{\text{U}}}_{n}$ with Eq. (15) or Eq. (16)

     **end for**

#### 2.2.1 Computation of point estimate $\hat{\theta }$ of forest parameters

Applying Bayes’s rule on Eq. (8) and taking logarithm, we obtain the following objective function:

(9) $$\begin{array}{c} \hat{\theta }=\underset{\theta }{\text{argmax}}\;p\left(\theta |\left\{M_{n}\right\},\left\{H_{n}\right\},\left\{K_{n}\right\},\left\{K_{n}^{h}\right\},\beta ,\gamma ,\sigma_{k}^{2}\right)\\ \;=\underset{\theta }{\text{argmax}}\;\text{log}\;p\left(\left\{K_{n}^{h}\right\},\left\{H_{n}\right\}|\theta ,\left\{M_{n}\right\},\left\{K_{n}\right\},\beta ,\gamma ,\sigma_{k}^{2}\right)+\text{log}\;p\left(\theta |\gamma \right). \end{array}$$

Exact maximisation of Eq. (9) would require marginalising over the deformation fields {***U**_n_*}, which leads to an intractable integral due to the pairwise terms of the MRF prior (Eq. (2)). Instead, we use a variational technique (VEM) for approximate inference. VEM inherits the advantages of standard EM optimisation (it does not require computing gradients or Hessian; it does not require tuning step sizes or backtracking; it is numerically stable; and it effectively handles hidden variables), while enabling (approximate) marginalisation over variables coupled by the MRF.

Since the Kullback–Leibler (KL) divergence is by definition non-negative, the objective function in Eq. (9) is bounded from below by:

(10) $$\begin{array}{l} J\left[q\left(\left\{{\text{U}}_{n}\right\}\right),\theta \right]=\text{log}\;p\left(\left\{K_{n}^{h}\right\},\left\{H_{n}\right\}|\theta ,\left\{M_{n}\right\},\left\{K_{n}\right\},\beta ,\gamma ,\sigma_{k}^{2}\}\right)\\ \;+\text{log}\;p\left(\theta |\gamma \right)\\ \;-KL[q\left(\left\{{\text{U}}_{n}\right\}\right)∥p(\left\{{\text{U}}_{n}\right\}|\left\{K_{n}^{h}\right\},\left\{H_{n}\right\},\theta ,\left\{M_{n}\right\},\\ \;\{K_{n}\},\beta ,\gamma ,\sigma_{k}^{2}\}) \end{array}$$

(11) $$\begin{array}{l} =\eta \left[q\right]+\sum_{\left\{U_{n}\right\}}q\left(\left\{{\text{U}}_{n}\right\}\right)\text{log}\;p\left(\left\{{\text{U}}_{n}\right\},\left\{K_{n}^{h}\right\},\left\{H_{n}\right\}|\theta ,\left\{M_{n}\right\},\left\{K_{n}\right\},\beta ,\gamma ,\sigma_{k}^{2}\}\right)\\ \;+\text{log}\;p\left(\theta |\gamma \right). \end{array}$$

The bound *J*[*q*({***U**_n_*}), *θ*] is the negative of the so-called free energy: *η* represents the entropy of a random variable; and *q*({***U**_n_*}) is a distribution over {***U**_n_*} which approximates the posterior $p\left(\left\{{\text{U}}_{n}\right\}|\left\{K_{n}^{h}\right\},\left\{H_{n}\right\},\theta ,\left\{M_{n}\right\},\left\{K_{n}\right\},\beta ,\gamma ,\sigma_{k}^{2}\}\right),$ while being restricted to have a simpler form. The standard mean field approximation (Parisi, 1988) assumes that *q* factorises over voxels for each field ***U**_n_*:

$$q\left(\left\{{\text{U}}_{n}\right\}\right)=\prod_{n=1}^{N}\prod_{\text{x}\in \Omega_{n}}q_{n\text{x}}\left({\text{U}}_{n}\left(\text{x}\right)\right),$$

where *q*_*n**x***_ is a discrete distribution over displacements at pixel ***x*** of image *n*, such that $q_{n\text{x}}\left(\Delta_{s}\right)\geq 0,\Sigma_{s=1}^{S}q_{n\text{x}}\left(\Delta_{s}\right)=1,\forall n,\text{x}.$

Rather than the original objective function (Eq. (9)), VEM maximises the lower bound *J*, by alternately optimising with respect to *q* (E-step) and *θ* (M-step) in a coordinate ascent scheme. We summarise these two steps below.

*E-step*. To optimise the lower bound with respect to *q*, it is convenient to work with Eq. (10). Since the first two terms are independent of *q*, one can minimise the KL divergence between *q* and the posterior distribution of {***U**_n_*} (see Equation S1 in the supplementary material). Building the Lagrangian (to ensure that *q* stays in the probability simplex) and setting derivatives to zero, we obtain:

(12) $$\begin{array}{l} q_{n\text{x}}\left(\Delta_{s}\right)\;\propto \;p\left(M_{n}\left(\text{x}+\Delta_{s}\right)|H_{n}\left(𝒲\left(\text{x}\right)\right),\theta \right)e^{-\beta_{1}∥\Delta_{s}{∥}^{2}}\\ \;\times \prod_{l=1}^{L_{n}}p{\left({\text{k}}_{nl}^{h}|{\text{k}}_{nl}-\Delta_{s},\sigma_{k}^{2}\right)}^{\delta \left({\text{k}}_{nl}=\text{x}\right)}\\ \;\times \text{exp}\left(\beta_{2}\sum_{{\text{x}}^{\prime }\in ℬ\left(\text{x}\right)}\sum_{s^{\prime }=1}^{S}{\left\|\Delta_{s}-\Delta_{s^{\prime }}\right\|}^{2}q_{n{\text{x}}^{\prime }}\left(\Delta_{s^{\prime }}\right)\right). \end{array}$$

**Fig. 2:**
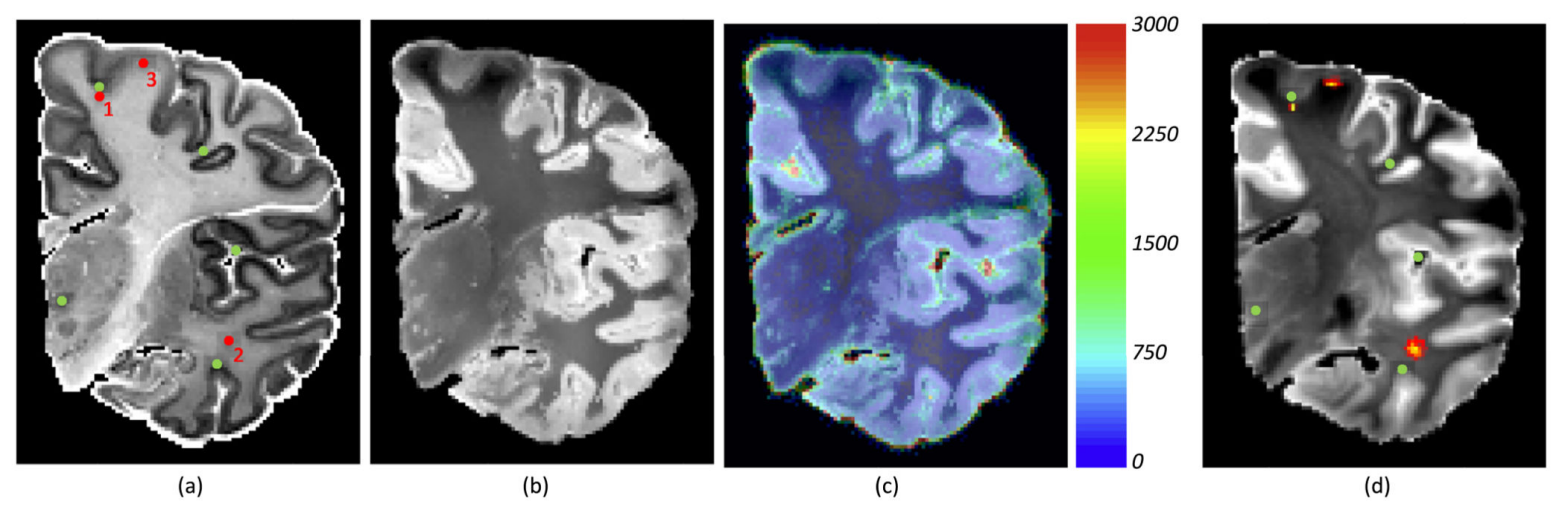
Uncertainty of registration and synthesis in the VEM algorithm: (a) Histological section from the Allen atlas. The green dots represent manually placed landmarks. (b,c) Mean and variance maps of the synthesised MRI slice, after 5 iterations of the VEM algorithm; higher variance corresponds to higher uncertainty in the synthesis. (d) Corresponding real MRI slice. The green dots represent the manually placed landmarks, corresponding to the ones in (a). The heat maps represent the variational distributions of displacements (*q_n**x**_*) corresponding to the red dots in (a), which illustrate the uncertainty in the registration. (For interpretation of the references to color in this figure legend, the reader is referred to the web version of this article.)

This equation has no closed-form solution, but can be solved with fixed point iterations, one image pair at the time – since there is no interdependence in *n*. We note that the effect of the landmarks is not local; in addition to creating a very sharp *q_n**x**_* around pixel at hand, the variational algorithm also creates a high confidence region around ***x***, by encouraging neighbouring pixels to have similar displacements. This user-informed, high-confidence region will have a higher weight in the synthesis, hence improving its quality. This effect is exemplified in Fig. 2(a,d), which illustrates the uncertainty in the two components (synthesis and registration) of the VEM algorithm. The spatial location marked by red dot number 1 is right below a manually placed landmark in the histological section, and the distribution *q_n**x**_* is hence strongly peaked at a location right below the corresponding landmark in the MRI slice. Red dot number 2, on the contrary, is located in the middle of the cerebral white matter, where there is little contrast to guide the registration, so *q_n**x**_* is much more spread and isotropic. Red dot number 3 lies in the white matter right under the cortex, so its distribution is elongated and parallel to the white matter surface.

*M-step*. When optimising *J* with respect to *θ*, it is more convenient to work with Eq. (11) – since the term *η*[*q*] can be neglected. Applying the chain rule of probability, and leaving aside terms independent of *θ*, we obtain:

(13) $$\begin{array}{l} \underset{\theta }{\;\text{argmax}}\sum_{\left\{{\text{U}}_{n}\right\}}q\left(\left\{{\text{U}}_{n}\right\}\right)\text{log}\;p\left(\left\{H_{n}\right\}|\left\{{\text{U}}_{n}\right\},\left\{M_{n}\right\},\theta \right)+\text{log}\;p\left(\theta |\gamma \right)\\ =\underset{\theta }{\text{argmax}}\sum_{n=1}^{N}\sum_{\text{x}\in \Omega_{n}}\sum_{s=1}^{S}q_{n\text{x}}\left(\Delta_{s}\right)\text{log}\;p\left(M_{n}\left(\text{x}+\Delta_{s}\right)|H_{n}\left(𝒲\left(\text{x}\right)\right),\theta \right)\\ \;+\text{log}\;p\left(\theta |\gamma \right). \end{array}$$

Maximisation of Eq. (13) amounts to training the regressor, such that each input image patch *H_n_* (𝒲(***x***)) is considered *S* times, each with an output intensity corresponding to a differently displaced pixel location *M_n_* (***x*** + **Δ**_*s*_), and with weight *q_n**x**_*(**Δ**_*s*_). In practice, and since injection of randomness is a crucial aspect of the training process of random forests, we found it beneficial to consider each patch *H_n_* (𝒲(***x***)) only once in each tree, with a displacement **Δ**_*s*_ sampled from the corresponding distribution *q_n**x**_*(**Δ**) – fed to the tree with weight 1.

The injection of additional randomness through sampling of **Δ** not only greatly increases the robustness of the regressor against misregistration, but also decreases the computational cost of training – since only a single displacement is considered per pixel. We also note that this sampling strategy still yields a valid stochastic optimiser for Eq. (13), since *q_n**x**_* is a discrete probability distribution over displacements. Such stochastic procedure (as well as other sources of randomness in the forest training algorithm) makes the maximisation of Eq. (13) only approximate; this means that the coordinate ascent algorithm to maximise the lower bound *J* of the objective function is no longer guaranteed to converge. In practice, however, the VEM algorithm typically converges after ~ 5 iterations.

Combined with the conjugate prior on the variance *p*(*θ*|*γ*), the joint prediction of the forest is finally given by:

(14) $$\begin{array}{l} \mu_{n\text{x}}=\frac{1}{T}\sum_{t=1}^{T}g_{t}\left[H_{n}\left(𝒲\left(\text{x}\right)\right);\theta \right]\\ \;\sigma_{n\text{x}}^{2}=\frac{2b+\sum_{t=1}^{T}{\left(g_{t}\left[H_{n}\left(𝒲\left(\text{x}\right)\right);\;\theta \right]-\mu_{n\text{x}}\right)}^{2}}{2a+T}, \end{array}$$

where *g_t_* is the guess made by tree *t*; *T* is the total number of trees in the forest; and where we have dropped the dependency of *μ_n**x**_* and *σ_n**x**_* on $\left\{H_{n},\hat{\theta }\right\}$ for simplicity.

Areas corrupted by artefacts lead to higher variances $\sigma_{n\text{x}\cdot }^{2}$ While the deformation model in our algorithm cannot describe cracks, holes or tears (which would require non-diffeomorphic deformation fields and an intensity model for missing tissue), our method copes well with these artefacts by yielding high uncertainty (variance) in these regions. This has the effect of decreasing the weight of these areas in the registration, as we will explain in Section 2.2.2 below. An example is shown in Fig. 2(b,c), in which the horizontal crack is assigned high uncertainty. High variance is also assigned to cerebrospinal fluid regions; while these areas do not display artefacts, their appearance might be bright or dark, depending on whether they are filled with paraformaldehyde, air or Fomblin (further details on these data can be found in Section 3.1).

#### 2.2.2 Computation of optimal deformation fields $\left\{{\hat{\text{U}}}_{n}\right\}$

Once the point estimate $\hat{\theta }$ (i.e., the optimal regression forest for synthesis) has been computed, one can obtain the optimal registrations by maximising $p\left(\left\{{\text{U}}_{n}\right\}|\hat{\theta },\left\{M_{n}\right\},\left\{H_{n}\right\},\left\{K_{n}\right\},\left\{K_{n}^{h}\right\},\beta ,\sigma_{k}^{2}\right).$ This amounts to maximising the log-posterior in Eq. (S2) in the supplementary material. Given the parameters, this posterior factorises over image pairs, and can thus be optimised on *n* at the time. Disregarding terms independent of ***U**_n_*, substituting the Gaussian likelihoods and switching signs in Equation S2 yields, for each image pair, the following cost function for the registration:

(15) $$\begin{array}{l} {\hat{\text{U}}}_{n}=\underset{{\text{U}}_{n}}{\text{argmin}}\underset{\text{Image}\;\text{term}}{\underset{︸}{\sum_{\text{x}\in \Omega_{n}}\frac{{\left[M_{n}\left(\text{x}+{\text{U}}_{n}\left(\text{x}\right)\right)-{\hat{\mu }}_{n\text{x}}\right]}^{2}}{2{\hat{\sigma }}_{n\text{x}}^{2}}}}\\ \;+\underset{\text{Landmark}\;\text{term}}{\underset{︸}{\frac{1}{2\sigma_{k}^{2}}\sum_{l=1}^{N_{l}}{\left\|{\text{k}}_{nl}^{h}-{\text{k}}_{nl}+{\text{U}}_{n}\left({\text{k}}_{nl}^{h}\right)\right\|}^{2}}}\\ \;+\underset{\text{Regularisation}}{\underset{︸}{\beta_{1}\sum_{\text{x}\in \Omega_{n}}{\left\|{\text{U}}_{n}\left(\text{x}\right))\right\|}^{2}+\beta_{2}\sum_{n=1}^{N}\sum_{\text{x}\in \Omega_{n}}\sum_{{\text{x}}^{\prime }\in ℬ\left(\text{x}\right)}{\left\|{\text{U}}_{n}\left(\text{x}\right)-{\text{U}}_{n}\left({\text{x}}^{\prime }\right)\right\|}^{2}}}, \end{array}$$

where the image term is a weighted sum of squared differences, in which the weights are inversely proportional to the variance of the forest predictions – hence downweighting the contribution of regions of high uncertainty in the synthesis. Thanks to the discrete nature of ***U**_n_*, a local minimum of the cost function in Eq. (15) can be efficiently found with algorithms based on graph cuts (Ahuja et al., 1993), such as Boykov et al. (2001).

We note that the result does not need to be diffeomorphic or invertible, which might be a desirable feature of the registration. This is due to the properties of the deformation model, which was chosen due to the fact that it easily enables marginalisation over the deformation fields with variational techniques. In practice, we have found that, once the optimal (probabilistic) synthesis has been computed, we can obtain smoother and more accurate solutions by using more sophisticated deformation models and priors. More specifically, we implemented the image and landmark terms of Eq. (15) in our registration package NiftyReg (Modat et al., 2010), instantly getting access to its advanced deformation models, regularisers and optimisers. NiftyReg parametrises the deformation field with a grid of control points combined with cubic B-Splines (Rueckert et al., 1999). If **Ψ**_*n*_ represents the vector of parameters of the spatial transform ***x***′ = ***V**(**x***; **Ψ***_n_*) for image pair *n*, we optimise:

(16) $$\begin{array}{l} {\hat{\Psi }}_{n}=\underset{\Psi_{n}}{\text{argmin}}\;\alpha \sum_{\text{x}\in \Omega_{n}}\frac{{\left[M_{n}\left(\text{V}\left(\text{x};\Psi_{n}\right)\right)-{\hat{\mu }}_{n\text{x}}\right]}^{2}}{2{\hat{\sigma }}_{n\text{x}}^{2}}\\ \;+\frac{1}{2\sigma_{k}^{2}}\sum_{l=1}^{N_{l}}{\left\|\text{V}\left({\text{k}}_{nl}^{h};\Psi_{n}\right)-{\text{k}}_{nl}\right\|}^{2}\\ \;+\beta_{b}E_{b}\left(\Psi_{n}\right)+\beta_{l}E_{l}\left(\Psi_{n}\right)+\beta_{j}E_{j}\left(\Psi_{n}\right), \end{array}$$

where *E_b_*(**Ψ**_*n*_) is the bending energy of the transform parametrised by **Ψ**_*n*_; *E_l_*(**Ψ**_*n*_) is the sum of squares of the symmetric part of the Jacobian after filtering out rotation (penalises stretching and shearing); *E_j_* is the Jacobian energy (given by its log-determinant); *β_b_* > 0, *β_l_* > 0, *β_j_* > 0 are the corresponding weights; and *α* > 0 is a constant that scales the contribution of the image term, such that it is approximately bounded by 1: *α*^−1^ = 9|Ω_*n*_|/2, i.e., a value of 1 is achieved if all pixels are three standard deviations away from the predicted mean.

Note that this choice for the final model also enables comparison with mutual information as implemented in NiftyReg, which minimises:

(17) $$\begin{array}{l} {\hat{\Psi }}_{n}^{MI}=\underset{{\text{U}}_{n}}{\text{argmin}}-\text{MI}\left[M_{n}\left(V\left(\text{x};\Psi_{n}\right)\right),H_{n}\left(x\right)\right]\\ \;+\frac{1}{2\sigma_{k}^{2}}\sum_{l=1}^{N_{l}}{\left\|\text{V}\left({\text{k}}_{nl}^{h};\Psi_{n}\right)-{\text{k}}_{nl}\right\|}^{2}\\ \;+\beta_{b}E_{b}\left(\Psi_{n}\right)+\beta_{l}E_{l}\left(\Psi_{n}\right)+\beta_{j}E_{j}\left(\Psi_{n}\right), \end{array}$$

where MI represents the mutual information. We note that finding the value of *α* that matches the importances of the data terms in Eqs. (16) and (17) is a non-trivial task; however, our choice of *α* defined above places the data terms in approximately the same range of values.

### 2.3 Summary of the algorithm and implementation details

The proposed method is summarised in Algorithm 1, and parameter settings (and criteria for setting them) are listed in Table 1. We define {**Δ_*s*_**} as a grid covering a square with radius 10 mm, in increments of 0.5 mm; this is enough to model all deformations we encountered in our datasets, since we assume that images are linearly pre-aligned. The approximate posteriors *q_n**x**_*(**Δ**) are initialised to 1/*S*, evenly spreading the probability mass across all possible displacements (i.e., maximum uncertainty in the registration). Given *q_n**x**_*, Eq. (13) is used to initialise the forest parameters *θ*. At that point, the VEM algorithm alternates between the E and M steps until convergence is reached. Convergence would ideally be assessed with *θ* but, since these parameters can vary significantly from one iteration to the next due to the randomness injected in training, we use the predicted means and variances instead $(\mu_{n\text{x}},\sigma_{n\text{x}}^{2}).$

In the E-step, each image pair can be considered independently. First, the histological section is pushed through the forest to generate a prediction for the (registered) MR image, including a mean and a standard deviation for each pixel (Eq. (14)). Then, fixed point iterations of Eq. (12) are run until convergence of *q_n**x**_*, ∀***x*** ∈ Ω_*n*_. In the M-step, the approximate posteriors *q* of all images are used together to retrain the random forest with Eq. (13). When the algorithm has converged, the final predictions (mean, variance) can be generated for each voxel, and the final registrations can be computed with Eq. (15), or with NiftyReg (see details below).

The random forest regressor used Gaussian derivatives (orders zero to three, and three scales: 0, 2 and 4 mm) and location as features. Injection of randomness is a crucial aspect of random forests, as it increases their generalization ability (Criminisi et al., 2011). Here we used bagging (Breiman, 1996) at both the image and pixel levels, and used random subsets of features when splitting data at the internal nodes of the trees. An additional random component in the stochastic optimization is the sampling of displacements **Δ** to make the model robust against misregistration (see Section 2.2.1). While all these random elements have beneficial effects, these come at the expense of giving up the theoretical guarantees on the convergence of the VEM algorithm – though this was never found to be a problem in practice, as explained in Section 2.2.1 above.

For the final registration, we used the default regularisation scheme in NiftyReg, which is a weighted combination of the bending energy (second derivative) and the sum of squares of the symmetric part of the Jacobian. We note that NiftyReg uses *β_j_* = 0 by default; while using *β_j_* > 0 guarantees that the output is diffeomorphic, the other two regularisation terms (*E_b_*, *E_l_*) ensure in practice that the deformation field is well behaved.

Table 1 summarises the values that we used for the parameters of the proposed algorithm, as well as those for the competing, mutual information based registration. We used a pilot image T1/T2 image pair to coarsely tune *β*_1_, based on visual inspection of the distributions *q_n**x**_* (i.e, as in Fig. 2d). We then heuristically set *β*_2_ = *β*_1_. All other parameters were set either heuristically or based on the default values from software packages, but never tuned on the data.

More specifically: we set the variance of the manual landmark placement to a low value, to reflect the high confidence in annotations provided by the user. We set the hyperparameters *γ* = [*a*, *b*]^T^ to values equivalent to a few (4) pseudo-observations with a small sample intensity variance (5^2^); the main objective is just to avoid pixels with zero variance in the synthesis. For the random forest, we used 100 trees. The more trees in the ensemble, the better the performance is expected to be – but the slower the training and testing are. The minimum number of samples in leaf nodes was set to 5, which is within the usual range in the literature (between 1 and 10). For the number of features sampled at each node in training, we used the square root of the total number of features, which is a common heuristic. The weight of the image term in Eq. 16 (*α*) attempted to match the range of this term to that of mutual information, with a value that makes it equal to 1 if all pixels are three standard deviations away from the mean predicted by the synthesis. Finally, all the parameters related to NiftyReg were set to the default values defined in the package, including the number of bins for computing the mutual information, and the relative weights of the different regularisers. The only parameter we swept in the experiments was the control point spacing of the final registration, which is well known to have a strong effect on the output.

## 3 Experiments and results

### 3.1 Data

We used three datasets to validate the proposed technique; two real (Allen Institute atlas, BigBrain atlas), and one synthetic. The real datasets enable us to assess how the algorithm behaves in a practical scenario. However, quantitative evaluation on real data is limited because it can only rely on manually placed landmarks, rather than full deformation fields – due to the unavailability of perfectly aligned histology-MRI data. For that reason, in addition to Allen and BigBrain, we have also included experiments on a synthetic MR dataset including T1-weighted and (synthetically deformed) T2-weighted scans. While these images are not necessarily an accurate substitute for the histology-MRI registration problem, they enable a direct, pixel-wise comparison of the estimated deformations with the ground truth fields that were used to generate them.

#### 3.1.1 Synthetic MRI dataset

The synthetic data were generated from 676 (real) pairs of T1- and T2-weighted scans from the publicly available ADNI dataset. The ADNI was launched in 2003 as a public-private partnership, led by Principal Investigator Michael W. Weiner, MD. The primary goal of ADNI has been to test whether serial magnetic resonance imaging, positron emission tomography, other biological markers, and clinical and neuropsychological assessment can be combined to measure the progression of mild cognitive impairment and early Alzheimers disease.

The resolution of the T1 scans was approximately 1 mm isotropic; the ADNI project spans multiple sites, different scanners were used to acquire the images; further details on the acquisition can be found at http://www.adni-info.org. The T2 scans correspond to an acquisition designed to study the hippocampus, and consist of 25–30 coronal images at 0.4 × 0.4 mm resolution, with slice thickness of 2 mm. These images cover a slab of tissue containing the hippocampi, which is manually oriented by the operator to be approximately orthogonal to the major axes of the hippocampi. Once more, further details on the acquisition at different sites can be found at the ADNI website.

The T1 scans were preprocessed with FreeSurfer (Fischl, 2012) in order to obtain skull-stripped, bias-field corrected images with a corresponding segmentation of brain structures (Fischl et al., 2002). We simplified this segmentation to three tissue types (gray matter, white matter, cerebrospinal fluid) and a generic background label. The processed T1 was rigidly registered to the corresponding T2 scan with mutual information, as implemented in NiftyReg (Modat et al., 2014). The registration was also used to propagate the brain mask and automated segmentation; the former was used to skull-strip the T2, and the latter for bias field correction using the technique described in Van Leemput et al. (1999). Note that we deform the T1 to the T2 – despite its lower resolution – because of its more isotropic voxel size.

From these pairs of preprocessed 3D scans, we generated a dataset of 1000 pairs of 2D images. To create each image pair, we followed these steps: 1. Randomly select one pair of 3D scans; 2. In the preprocessed T2 scan, randomly select a (coronal) slice, other than the first and the last, which sometimes display artefacts; 3. Downsample the T2 slice to 1 × 1 mm resolution, for consistency with the resolution of the T1 scans; 4. Reslice the (preprocessed) T1 scan to obtain the 2D image corresponding to the downsampled T2 slice; 5. Sample a random diffeomorphic deformation field (details below) in the space of the 2D slice; 6. Combine the deformation field with a random similarity transform, including rotation, scaling and translation; 7. Deform the T2 scan with the composed field (linear + nonlinear). 8. Rescale intensities to [0,255] and discretise with 8-bit precision. Note than we deform the T2 slices – rather than the T1 counterpart – to avoid interpolating the T1 data twice. The T2 images play the role of the MRI, and the T1s play the role of histology.

**Fig. 3:**
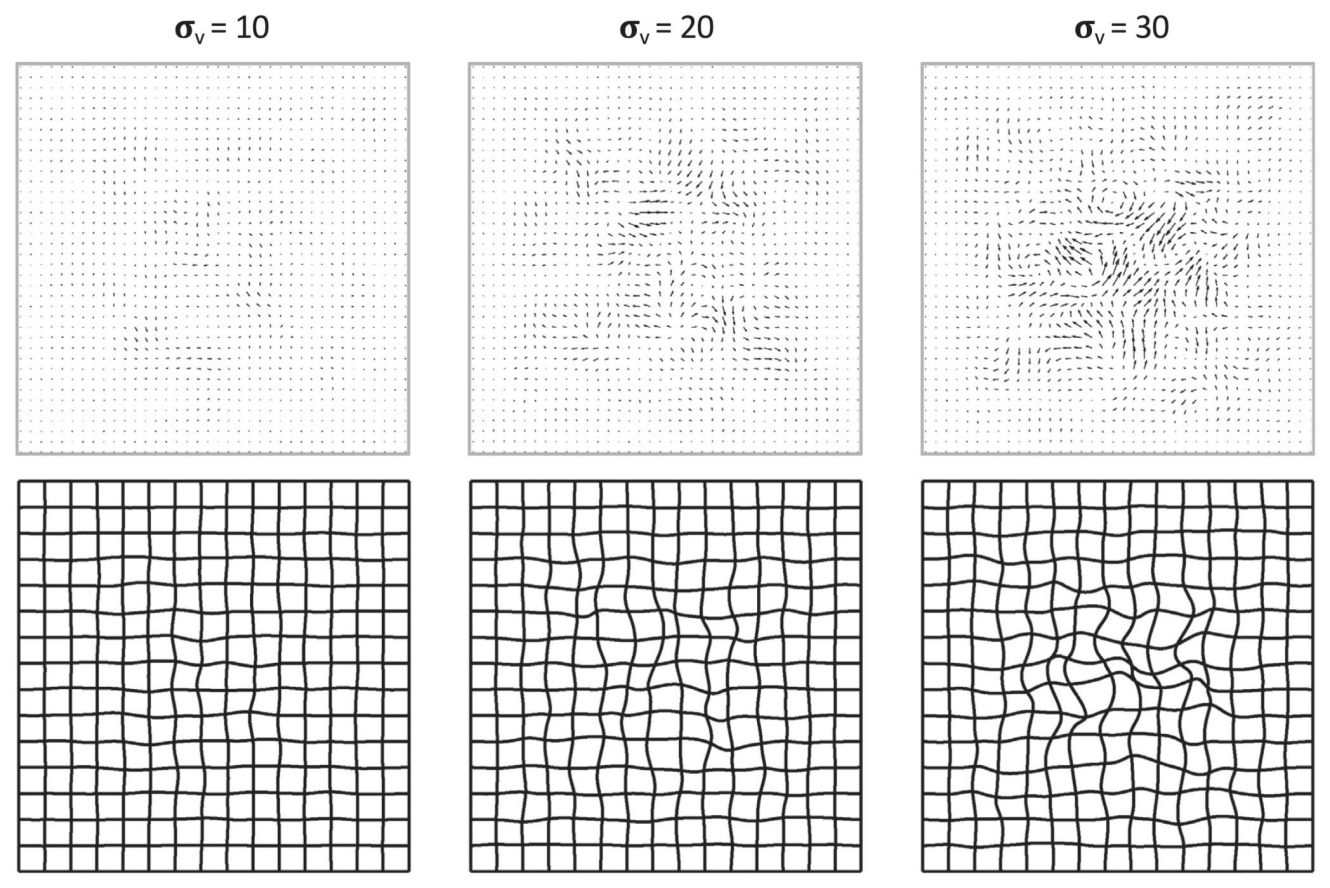
Synthetic velocity (top row) and corresponding deformation fields (bottom row) generated with three different levels of noise *σ_v_*.

To generate synthetic fields without biasing the evaluation, we used a deformation model different from that used by NiftyReg (i.e., a grid of control points and cubic B-Splines). More specifically, we created diffeormorphic deformations as follows. First, we generated random velocity fields by independently sampling bivariate Gaussian noise at each spatial location (no x-y correlation) with different levels of variance; smoothing them with a Gaussian filter; and multiplying them by a window function in order to prevent deformations close to the boundaries; we used exp [0.01*D*(***x***)], where *D*(***x***) is the distance to the boundary of the image in mm. Then, these velocity fields were integrated over unit time using a scaling and squaring approach (Moler and Van Loan, 2003; Arsigny et al., 2006) to generate the deformation fields. Sample velocity and deformation fields generated with different levels of noise are shown in Fig. 3.

Given the synthetic deformation fields, we generated spatially spread pairs of salient landmarks with the following iterative procedure: 1. Feeding the T1 slice through a Harris corner detector (Harris and Stephens, 1988). 2. Taking the pixel with the highest response ***x**_max_*, following the ground truth deformation to obtain the corresponding location in the deformed T2 slice, and corrupting it with Gaussian noise of variance $\sigma_{k}^{2};$ this pair of locations is added to the set of landmarks of the slice. 3. Multiplying the Harris response by a complementary Gaussian function centred at ***x**_max_*, i.e., *f*(***x***) = 1 − exp[−0.5‖***x*** − ***x**_max_*‖^2^/*σ*^2^], with standard deviation *σ* equal to 1/10 of the image dimensions; this ensures that the following landmarks will be far from the current ***x**_max_*, eventually leading to a set of spatially spread set. 4. Going back to Step 2, until enough landmarks have been generated. In this iterative procedure, the Harris detector ensures that landmarks are located at salient points (rather than image regions of flat appearance), mimicking the way in which human labellers place landmarks. The complementary Gaussian, on the other hand, ensures that the landmarks are spatially distributed across the images, in order to assist the registration across the full image domain. This automated landmark generation procedure is illustrated in the example in Fig. 7b.

#### 3.1.2 Real data: Allen dataset

The Allen atlas is based on the left hemisphere of a 34-year-old donor. The histology of the atlas includes 106 Nissl-stained sections of the whole hemisphere in coronal plane, with manual segmentations of 862 brain structures. Sample sections of the dataset are shown in Figs. S7 and S8 of the supplementary material. Due to the challenges associated with sectioning and mounting thin sections from complete hemispheres, artefacts such as holes, large cracks, and severe staining inhomogeneities are prevalent in this dataset; see examples in Figure S8, or the horizontal crack in Fig. 2a. These artefacts make the Allen atlas representative of typical histological images, and hamper image registration.

The sections of the Allen atlas are 50 μm thick, and digitised at 1 μm in-plane resolution with a customised microscopy system – though we downsampled them to 200 μm to match the resolution of the MRI data (details below). We also downsampled the manual segmentations to the same resolution, and merged them into a whole brain segmentation that, after dilation, we used to mask the histological sections. The histology and associated segmentations can be interactively visualised at http://atlas.brain-map.org, and further details can be found in Ding et al. (2016). No 3D reconstruction of the histology was performed in their study.

In addition to the histology, high-resolution MRI images of the whole brain were acquired on a 7 T Siemens scanner with a custom 30-channel receive-array coil. The specimen was scanned in a vacuum-sealed bag surrounded by Fomblin to avoid artefacts caused by air-tissue interfaces. The images were acquired with a multiecho flash sequence (TR = 50 ms; *α* = 20°, 40°, 60°, 80°; echoes at 5.5, 12.8, 20.2, 27.6, 35.2, and 42.8 ms), at 200 μm isotropic resolution. Once more, the details can be found in Ding et al. (2016). In this study, we used a single volume, obtained by averaging the echoes corresponding to flip angle *α* = 20°, which provided good contrast between gray and white matter tissue, as well as great signal-to-noise ratio. The combined image was bias field corrected with the method described in Van Leemput et al. (1999) using the probability maps from the LONI atlas (Shattuck et al., 2008), which was linearly registered with NiftyReg (Modat et al., 2014). A coarse mask for the left hemisphere was manually delineated by JEI, and used to mask out tissue from the right hemisphere, which is not included in the histological analysis. Sample coronal slices of this dataset are shown in Fig. 2a (histology) and b (MRI).

#### 3.1.3 Real data: BigBrain dataset

The publicly available BigBrain atlas consists of a full brain of a 64-year-old donor (Amunts et al., 2013). The brain was embedded in paraffin and, using a large-scale microtome, cut into 7404 coronal sections with 20 μm thickness. All 7404 sections were stained for cell bodies, and digitised at 20 μm resolution – to match the section thickness. Sample sections of the dataset are shown in Figs. S9 and S10 of the supplementary material. As in the Allen Atlas, severe artefacts (though not as pronounced) are prevalent in this dataset – see examples in Fig. S10. The atlas also includes an MRI scan of the sample, which was acquired on a 1.5T scanner using a 12-channel coil. The volume was acquired with an MPRAGE sequence with parameters: TR = 2220 ms, TE = 3 ms, IR = 1200 ms, *α* = 15°, resolution 0.4 × 0.4 × 0.8 mm^3^, 6 averages. The sample was scanned inside a Plexiglas cylinder and kept in formalin; extensive degassing of the formalin was performed to eliminate air bubbles. No manual segmentations are available for this dataset.

In addition to the raw data, the BigBrain dataset includes a very accurate 3D reconstruction of the histology, which was performed with a complex pipeline that involved not only manual intervention, but also ~ 250, 000 h of CPU time on a high-performance computing cluster (see details in Amunts et al., 2013). Since Big-Brain provides an approximate spatial alignment between the MRI scan and the 3D reconstruction of the histology, it is straightforward to derive a correspondence between histological sections and corresponding coronal slices. Therefore, rather than using the full raw dataset (placing manual landmarks on 7404 pairs of images would be excruciating), we only considered the histological sections that correspond to coronal slices in the brain MRI scan. We left aside the first and last 20 slices, which contain very little tissue, ending up with 331 pairs of images (histological sections and MRI slices). We downsampled these histological sections to 400 *μ*m pixel size, to match the resolution of the MRI.

### 3.2 Experimental setup

In the experiments, we compared the performance of our proposed method with that of mutual information based registration. First, we conducted thorough experiments on the synthetic data, in which we swept the control point spacing in the registration. And second, we used the optimal parameter settings to register the real data from the Allen Institute and the BigBrain atlas. We note that we use the NiftyReg implementation of mutual information based registration as competing method, because it is the only way of comparing the image terms of the two approaches (i.e., Eqs. (16) and (17)) in a fair manner. In other words: if we used a different registration package as competing method, we could not disambiguate whether differences in performance stem from the image terms or from differences in implementation details, regularisers, etc.

In the synthetic data, we considered three different levels of Gaussian noise (*σ_v_* = 10, 20, 30 mm) when generating the velocity fields, in order to model nonlinear deformations of different severity. The standard deviation of the Gaussian smoothing filter was set to 5 mm, in both the horizontal and vertical direction. The random rotations, translations and log-scalings of the similarity transform were sampled from zero-mean Gaussian distributions, with standard deviations of 2°, 1 pixel, and 0.1, respectively. We then used NiftyReg with mutual information and our method to recover the deformations, both using the same landmark sets. We used different spacings between control points (from 3 to 21 mm, with 3 mm steps) to evaluate different levels of model flexibility. Otherwise we used the parameters listed in Table 1, both for our proposed method (Eq. (16)) and mutual information (Eq. (17)). We tested our algorithm in two different scenarios: running it on all image pairs simultaneously, or on each image pair independently (i.e., with *N* = 1). In the former case, bagging was used at both the slice and pixel levels, using 66% of the available images, and as many pixels per image as necessary in order to have a total of 25,000 training pixels. In the latter case, which represents the common case that a user runs the algorithm on just a pair of images, we used 66% of the pixels to train each tree.

In the Allen Institute data, we compared mutual information based registration with our approach, using all slices simultaneously in the synthesis with bagging (as for the synthetic data, using 66% of the images in each tree, randomly sampling 25,000 pixels). In order to put the MRI in linear alignment with the histological sections, we used an iterative approach very similar to that of Yang et al. (2012). Starting from a stack of histological sections, we first rigidly aligned the brain MRI to the stack using mutual information. Then, we resampled the registered MRI to the space of each histological section, and aligned them one by one using a similarity transform combined with mutual information. The registration of the MRI was then refined using the realigned sections, starting a new iteration. Upon convergence of the linear registration procedure, we resampled the MR images into the space of the histological sections. Next, a human labeller (JEI) manually annotated 1104 pairs of landmarks – approximately 11 per image pair. The landmarks were placed on salient points that were easy to recognise on both images (e.g., corners of sulci, gyri, and subcortical structures), while being spatially spread across the images – in order to inform the registration throughout the whole image domain. The exact number of landmarks on each image pair depends on the amount of tissue in the histological section, and the observer’s discretion. These landmarks were randomly divided into two folds, with cross-validation purposes. We then used the two competing methods to nonlinearly register the histological sections to the corresponding resampled MR images. We used the same parameters as for the experiment with the synthetic data, setting the control point spacing to the optimal values from such experiments (6 mm for the proposed approach, and 18 mm for mutual information; see Section 3.3.1 below); note that, for the manual landmarks, *σ_k_* = 0.5 mm was equivalent to 2.5 pixels at the resolution of this dataset – rather than one pixel, as in the synthetic data. We produced three different registrations with each method: one using all landmarks (for qualitative evaluation based on visual inspection), and two using the landmarks in the cross-validation folds (for quantitative evaluation).

Finally, the experimental setup for the BigBrain data was almost the same as for the Allen Institute data. Again, we compared our approach with mutual information based registration. The parameters for the synthesis was the same as for Allen. We note that it was not necessary to rigidly align the MRI to the histology, as an approximate alignment is already given in this dataset, as explained in Section 3.1.3. As for the Allen dataset, JEI manually labelled 3, 839 pairs of landmarks across the 331 image pairs (approximately 12 per pair, placed on salient points), which were randomly split into two folds. The control point spacing was again 6 mm for the proposed approach and 18 mm for mutual information. Once more, we computed registrations using all the landmarks, for qualitative evaluation, but also using the landmarks within each fold, for quantitative evaluation. In this dataset, *σ_k_* = 0.5 mm was equivalent to 1.25 pixels.

### 3.3 Results

#### 3.3.1 Synthetic data

**Fig. 4:**
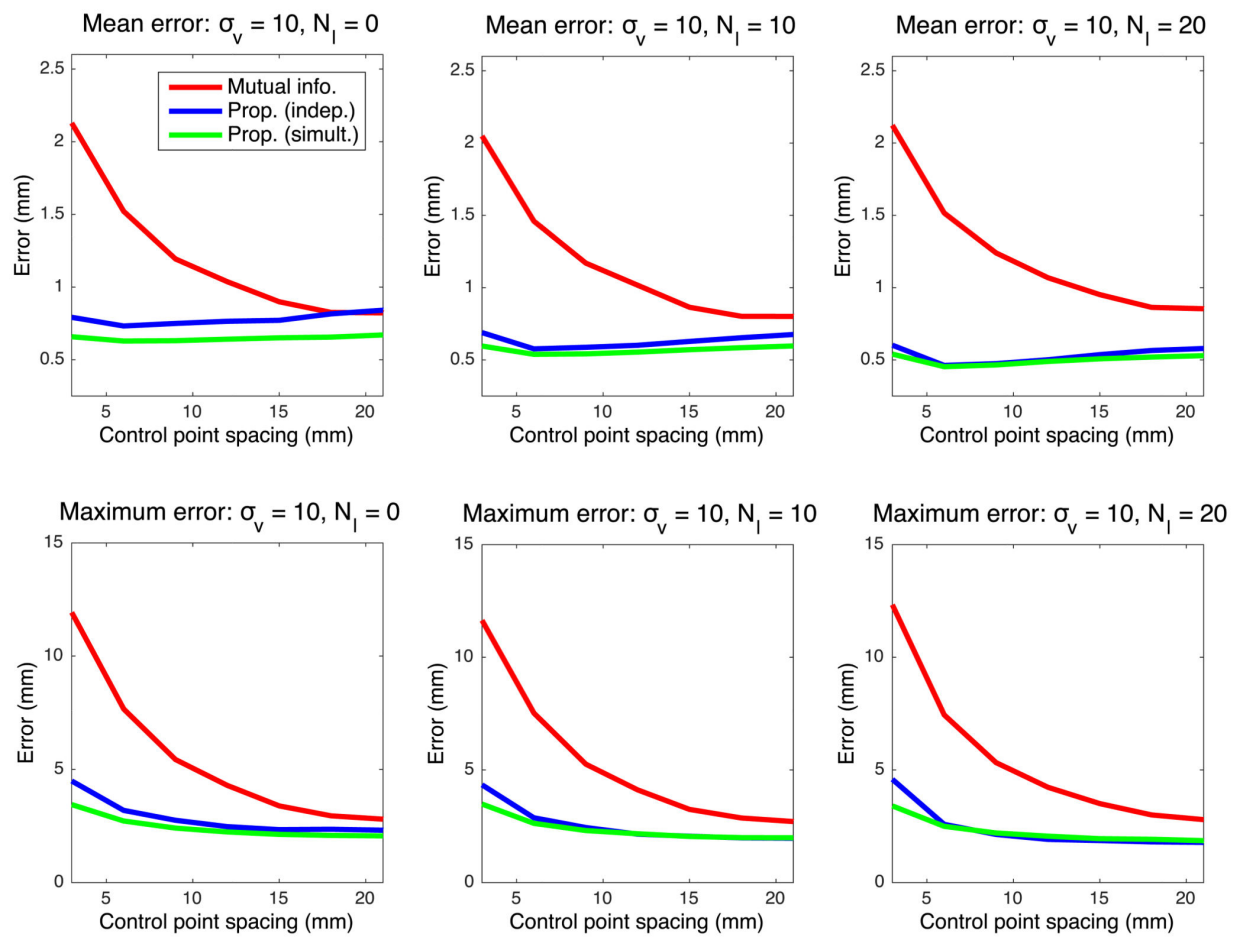
Mean and maximum registration error in mm for deformations with *σ_v_* = 10 (mild).

**Fig. 5:**
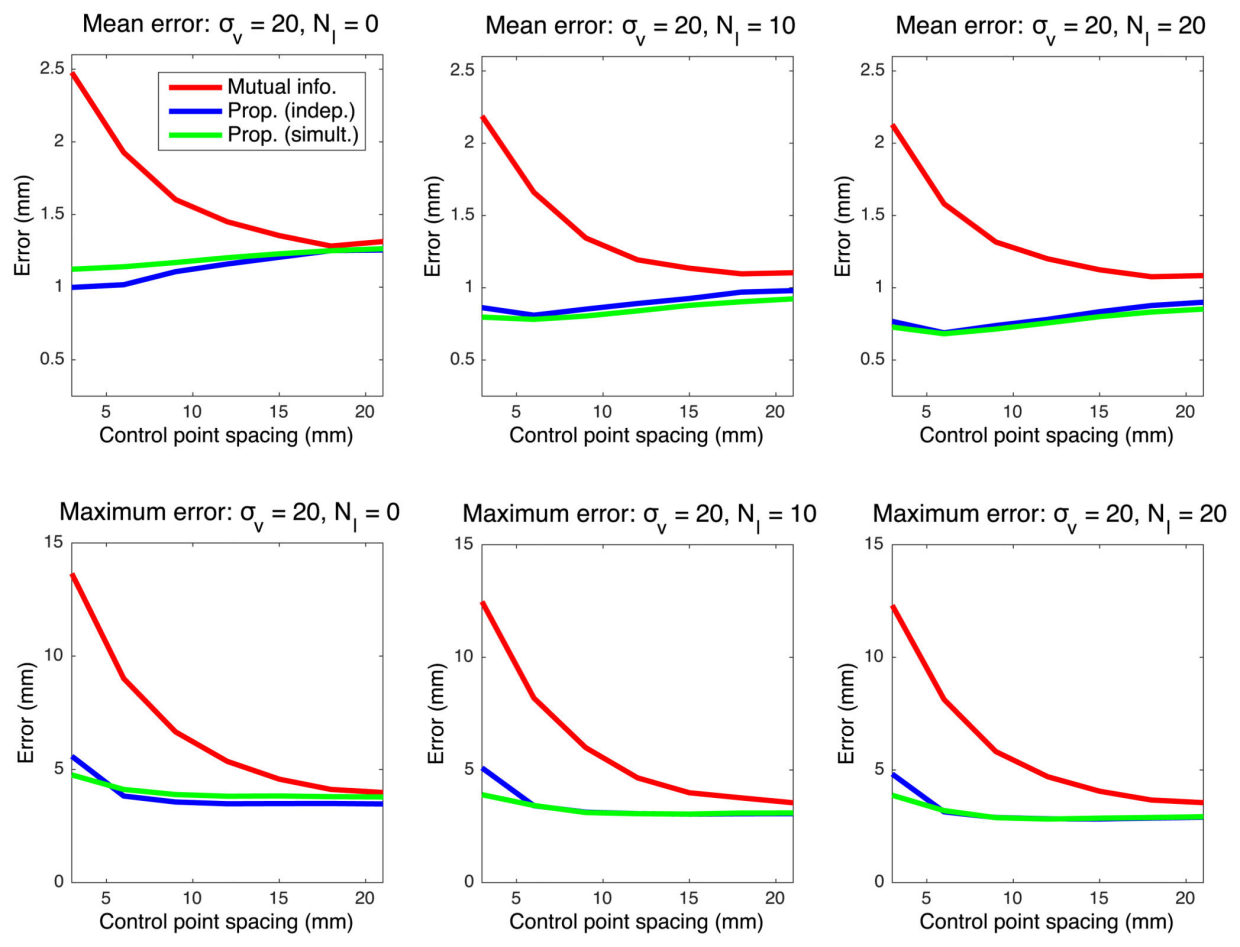
Mean and maximum registration error in mm for deformations with *σ_v_* = 20 (medium).

**Fig. 6:**
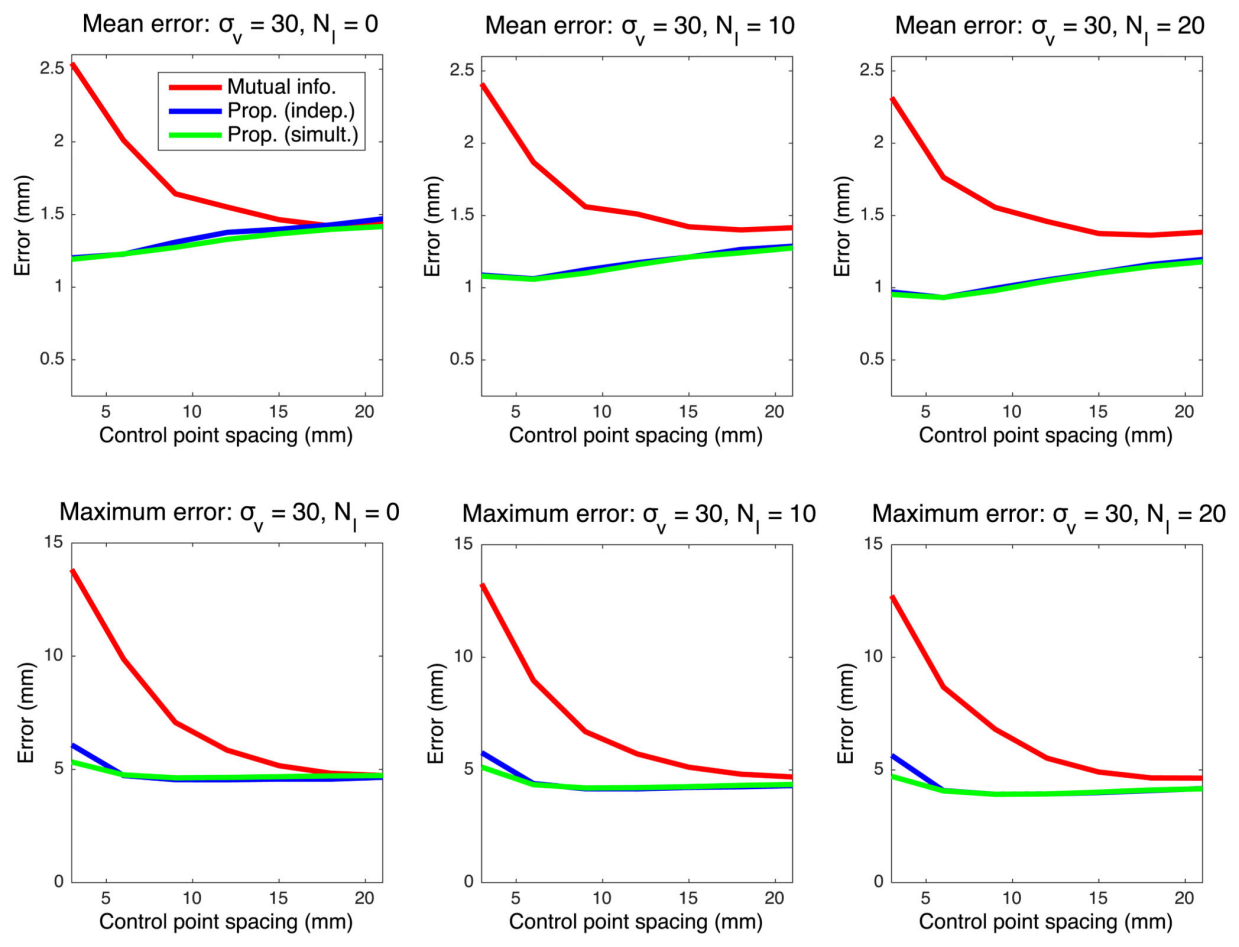
Mean and maximum registration error in mm for deformations with *σ_v_* = 10 (strong).

**Fig. 7:**
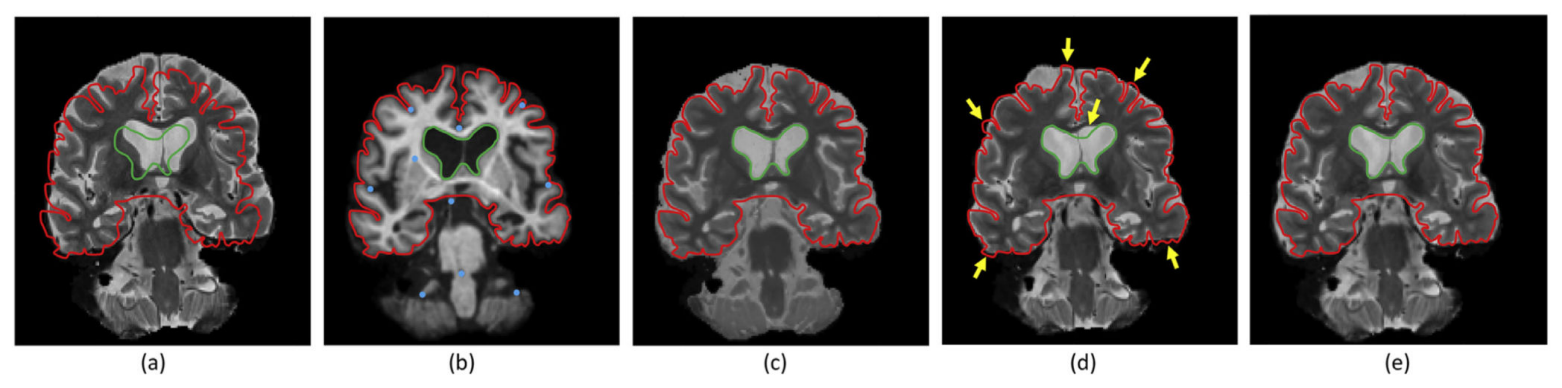
Example from synthetic dataset: (a) Deformed T2 image, used as floating image in the registration. (b) Corresponding T1 scan, used as reference image, with 10 automatically placed landmarks (blue dots) overlaid. (c) Corresponding synthetic T2 image, after 5 iterations of our VEM algorithm. (d) Registered with mutual information. (e) Registered with our algorithm. Both in (d) and (e), the control point spacing was set to 6 mm. We have overlaid on all five images a manual outline of the gray matter surface (in red) and of the ventricles (in green), which were drawn using the T1 scan (b) as a reference. Note the poor registration produced by mutual information in the ventricles and cortical regions – see for instance the areas pointed by the yellow arrows in (d). (For interpretation of the references to color in this figure legend, the reader is referred to the web version of this article.)

Figs. 4–6 show the mean registration error as a function of the control point separation and the number of landmarks for three different levels of noise deformation: 10, 20 and 30 mm, which correspond to mild, medium and strong deformations, respectively. The mean error reflects the precision of the estimation, whereas the maximum is related to its robustness. When using mutual information, finer control point spacings in the deformation model yield transforms that are too flexible, leading to very poor results (even in presence of control points); see example in Fig. 7. Both the mean and maximum error improve with larger spacings, flattening out at around 18–20 mm.

The proposed method, on the other hand, provides higher precision with flexible models, thanks to the higher robustness of the intramodality metric. The two versions of the method (estimating the regressor one image pair at the time or from all images simultaneously) consistently outperform mutual information in every scenario. An important difference in the results is that the mean error hits its minimum at a much smaller control point spacing (typically 6 mm), yielding a much more accurate registration; see example in Fig. 7, and also further examples – including orthogonal views (i.e., from 3D reconstructions) – in Figs. S1-S6 in the supplementary material. Moreover, the maximum error has already flattened at that point (6 mm) in almost every tested setting.

In addition to supporting finer control points spacings, the proposed method can more effectively exploit the information provided by landmarks. In mutual information based registrations, the landmarks guide the registration, especially in the earlier iterations, since their relative cost is high. However, the landmarks only constrain the deformation field locally, and further influence on the registration (e.g., by improving the estimation of the joint histogram) is indirect and very limited. Therefore, the quantitative effect of adding landmarks on the mean and maximum errors is rather small.

Our proposed algorithm, on the other hand, explicitly exploits the landmark information not only in the registration, but also in the synthesis. Following the exponential MRF term in Eq. (12), the landmarks sharpen the distribution *q* not only at their locations, but also in their surroundings (see for instance Tag 1 in Fig. 2d). Therefore, very similar displaced locations of these pixels are consistently selected when sampling for each tree of the forest, which greatly informs the learning of the appearance model, i.e., the synthesis – particularly since the model is learned directly from the test data, and adapts to variations in staining, MRI contrast, etc. Increased number of landmarks *N_l_* yields higher performance both for our proposed method and mutual information. However, given that better synthesis leads to improved registration, the gap in performance between the two methods actually widens as *N_l_* increases, as reflected by the quantitative results.

When no landmarks are used and image pairs are assessed independently, the proposed algorithm can be seen as a conventional inter-modality registration method. In that scenario, the results discussed above still hold: our method can be used at finer control point spacings, and provides average reductions of 11%, 22% and 15% in the mean error, at *σ_v_* = 10, *σ_v_* = 20 and *σ_v_* = 30, respectively. We also note that, as one would expect, our method and mutual information produce almost identical results at large control point spacings.

Finally, we note a modest improvement is observed when image pairs are considered simultaneously – rather than independently. Nevertheless, the joint estimation consistently yields higher robustness at the finest control point spacing (3 mm), and also produces smaller errors across the different settings when the deformations are mild (Fig. 4). We hypothesise that, even though the simultaneous estimation has the advantage of having access to more data (which is particularly useful with more flexible models, i.e., finer spacing), the independent version can also benefit from having a regressor that is tailored to the single image pair at hand.

#### 3.3.2 Results on Allen Institute data

Table 2 displays the quantitative results for this dataset. In absolute terms, the errors are larger than for the synthetic data in Section 3.3.1 above, due to the starker differences in image contrast between the two modalities, and the presence of artefacts in the histology. Still, the proposed method provides a significant (*p* ~ 10^−33^) reduction in registration error, compared with the baseline, mutual information based approach; we note that registration errors are not independent across landmarks or even images, so statistical testing produces underestimated p values, but the results still clearly point towards a statistically significant improvement.

**Fig. 8:**
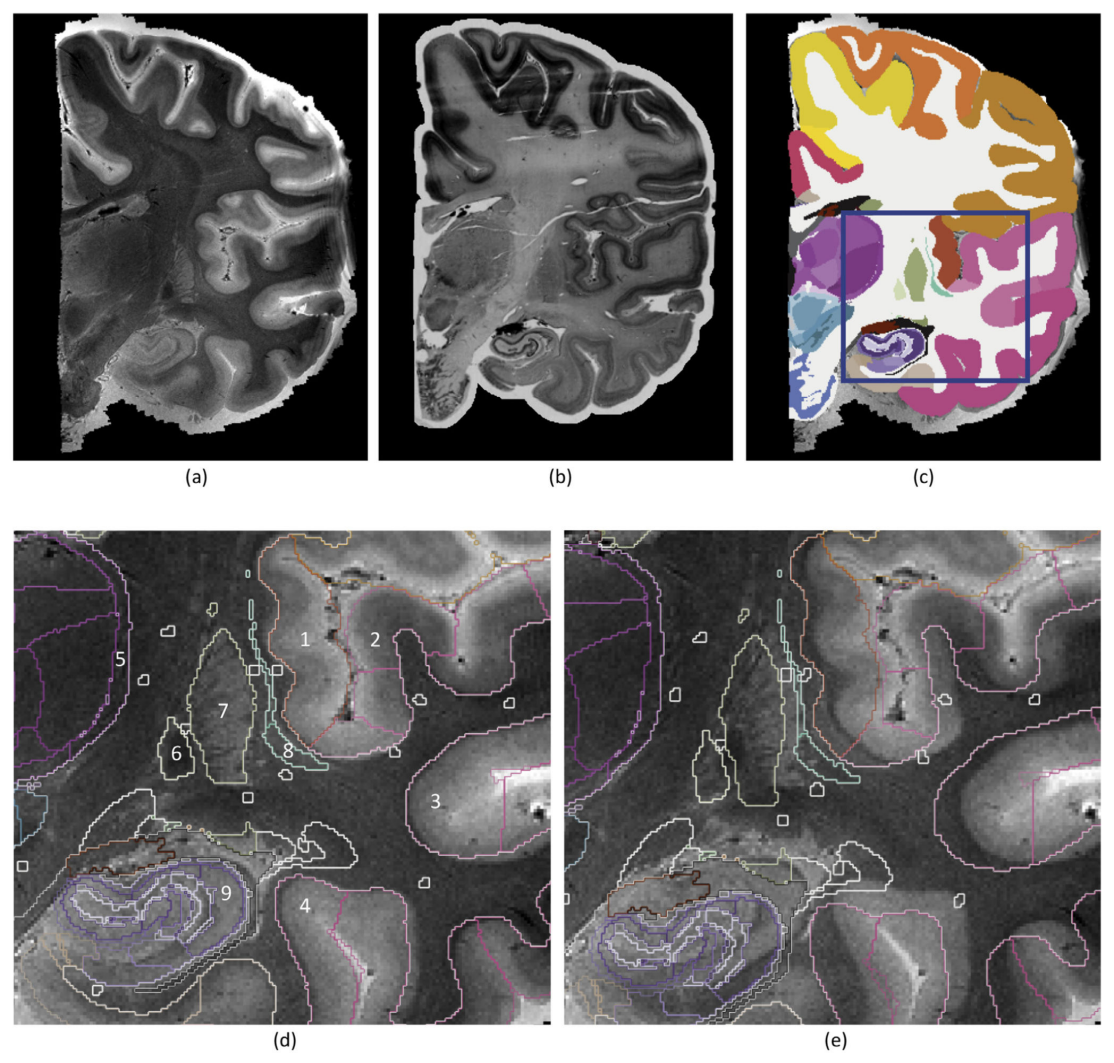
(a) Coronal slice of the MRI scan. (b) Corresponding histological section, registered with the proposed method. (c) Corresponding manual segmentation, propagated to MR space. (d) Close-up of the region inside the blue square, showing the boundaries of the segmentation; see main text (Section /3.3.2) for an explanation of the numerical tags. (e) Segmentation obtained when using mutual information in the registration. See http://atlas.brain-map.org for the color map. (For interpretation of the references to color in this figure legend, the reader is referred to the web version of this article.)

The decrease in registration error is also apparent from the registered images. Fig. 8 shows a representative coronal section of the data, which covers multiple cortical and subcortical structures of interest (e.g., hippocampus, thalamus, putamen and pallidum). Comparing the segmentations propagated from the histology to the MRI with the proposed method (Fig. 8d) and mutual information (Fig. 8e) using all available landmarks in both cases, it is apparent that our algorithm produces a much more accurate registration. The contours of the white matter surface are rather inaccurate when using mutual information; see for instance the insular (Tag 1 in the figure), auditory (Tag 2), or polysensoral temporal cortices (Tag 3); or area 36 (Tag 4). Using the proposed method, the registered contours follow the underlying MRI intensities much more accurately. The same applies to subcortical structures. In the thalamus (light purple), it can be seen that the segmentation of the reticular nucleus (Tag 5) is too medial when using mutual information. The same applies to the pallidum (Tag 6), putamen (Tag 7) and claustrum (Tag 8). The hippocampus (dark purple; Tag 9) is too inferior to the actual anatomy in the MRI. Once more, the proposed algorithm produces, qualitatively speaking, much improved boundaries.

**Fig. 9:**
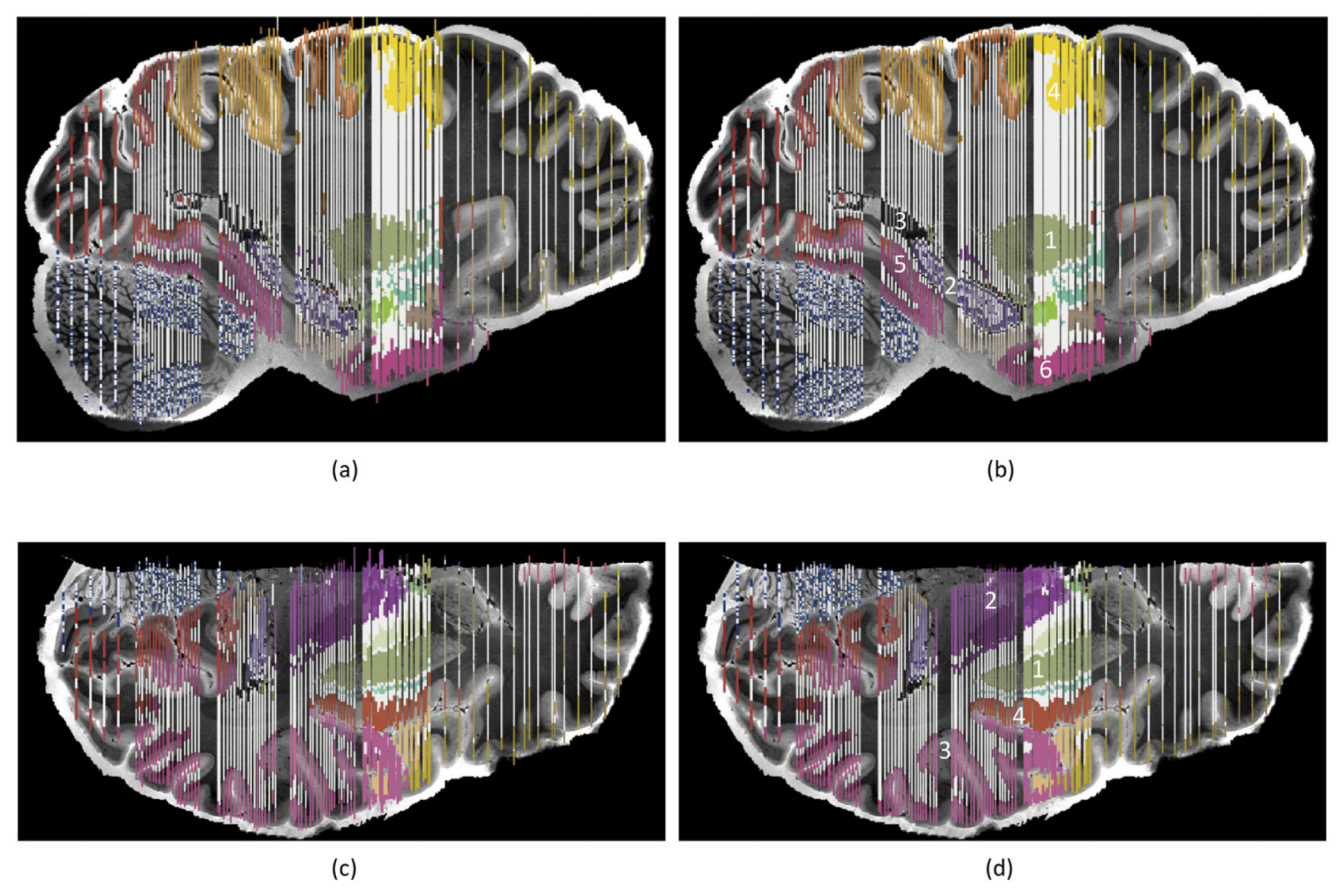
(a) Sagittal slice of the MRI scan, with registered segmentation superimposed. The deformation fields used to propagate the manual segmentations from histology to MRI space were computed with mutual information. (b) Same as (a), but using our technique to register the data. (c) Axial slice, reconstruction with mutual information. (d) Same slice, reconstructed with our proposed method. See http://atlas.brain-map.org for the color map.

To better assess the quality of the reconstruction as a whole (rather than on a single slice), Fig. 9 shows the propagated segmentations in the orthogonal views: sagittal (Fig. 9a, b) and axial (Fig. 9c, d). The proposed method produces reconstructed segmentations that are smoother and that better follow the anatomy in the MRI scan. In sagittal view, this can be easily observed in subcortical regions such as the putamen (Tag 1 in Fig. 9b), the hippocampus (Tag 2) or the lateral ventricle (Tag 3); and also in cortical regions such as the premotor (Tag 4), parahippocampal (Tag 5) or fusiform temporal (Tag 6) cortices. The improvement is also apparent from how much less frequently the segmentation leaks outside the brain when using our algorithm. Similar conclusions can be derived from the axial view; see for instance the putamen (Tag 1 in Fig. 9d), thalamus (purple region, Tag 2), polysensory temporal cortex (Tag 3) or insular cortex (Tag 4).

#### 3.3.3 Results on BigBrain data

Table 3 displays the quantitative results for the BigBrain dataset. The errors are once more clearly larger than for the synthetic dataset, but slightly smaller than for the Allen Institute data, since the artefacts are not as strong in this dataset (e.g., compare Figure S8 with Figure S10). As in Section 3.3.1, our method provides a significant improvement over mutual information based registration (*p* ~ 10^−23^), with reduced mean, median and maximum registration errors (again, p values need to be interpreted with caution due to the lack of statistical independence between landmarks and images).

**Fig. 10:**
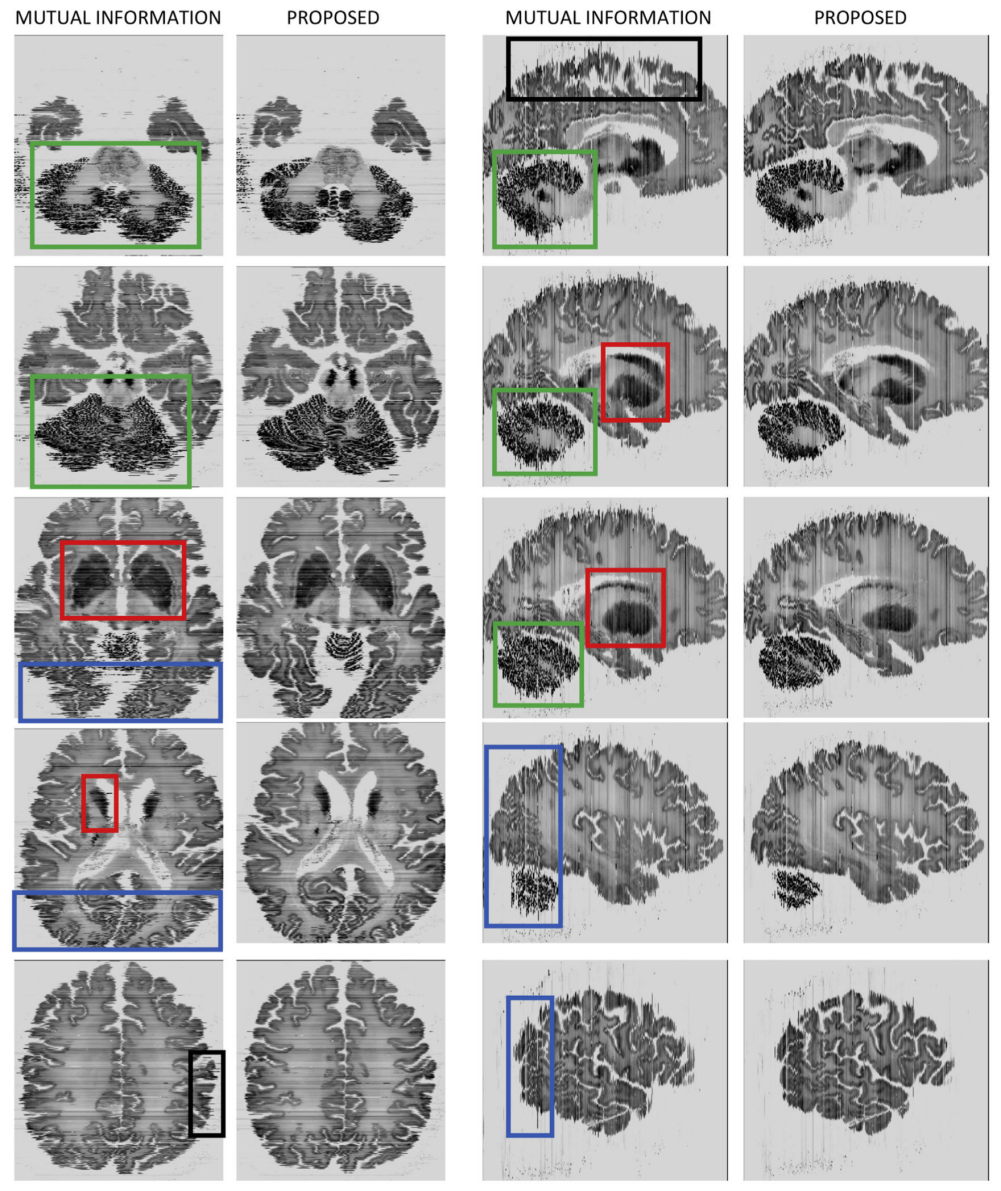
Orthogonal views of reconstructed BigBrain using mutual information based information and our approach. Leftmost columns: axial view, from inferior to superior. Rightmost columns: sagittal view, from medial to lateral. The boxes mark areas in which the proposed method yields more accurate results than mutual information based registration; please see text in Section 3.3.3 for explanations.

Fig. 10 shows qualitative results for this dataset. More specifically, the figure displays a set of reconstructed slices in the two planes orthogonal to the sectioning direction, i.e., axial and sagittal. The proposed method yields reconstructions that are more consistent than those produced by mutual information. Areas that are clearly better reconstructed include: the cerebellum, for which the reconstruction is crisper in every slice in which it is visible (see green boxes in the figure); the basal ganglia, which is greatly and artificially enlarged by mutual information based registration (see red boxes); the occipital region, in which our proposed method yields a much smoother reconstruction (see blue boxes in the figure); and the cortical surface, which is smoother when reconstructed with our method in all images in the figure (see for example the areas marked with black boxes). Finally, we note that these reconstructions are not as sharp as those in the BigBrain website; it is not our goal here to produce reconstructions of such high quality, which would require careful artefact correction, intensity normalisation, and considering the intra-modality registration of neighbouring sections in the reconstruction.

## 4 Discussion and conclusion

In this article, we presented a novel method to simultaneously estimate the registration and synthesis between a pair of corresponding images from different modalities. The results on both synthetic and real data show that the proposed algorithm is superior to standard inter-modality registration based on mutual information, albeit slower due to the need to iterate between registration and synthesis – especially the former, since it requires nested iteration of Eq. (12). Our Matlab implementation runs in 2–3min for images of size 256^2^ pixels, but parallelised implementation in C++ or on the GPU should greatly reduce the running time.

The quantitative experiments on synthetic data demonstrated that our algorithm supports much more flexible deformation models than mutual information (i.e., smaller control point spacing) without compromising robustness, attributed to the more stable intra-modality metric (which we have made publicly available in NiftyReg). Moreover, these experiments also showed that our algorithm can more effectively take advantage of the information encoded in manually placed pairs of landmarks. Mutual information alone only benefits from the constraints that landmarks introduce in the deformation fields, which yields a small decrease in registration error. Our method, on the other hand, also exploits landmark information in synthesis, which further improves the results, as registration and synthesis inform each other in model fitting. The more landmarks we used, the larger the gap between our method and mutual information was – however, we should note that, in the limit, the performance of the two methods would be the same, since the registration error would go to zero in both cases.

The proposed method relies on a number of parameters, which influence the final result. As explained in Section 2.3, these parameters were set to sensible values defined *a priori*, except for the parameters of the MRF, which were coarsely tuned by visual inspection of the output on a pilot dataset. The fact that the same parameter values produced satisfactory outputs in all three datasets indicates that the output is not too sensitive to parameter settings. The only parameter that has a great influence on the results is the control point spacing – which is well known from the image registration literature. This is the reason why control point spacing is the only parameter – along with landmark count – that we swept in the experiments to find suitable values. On a related note, we must note that, in the experiments with synthetic data, the relative contributions of the data terms in Eqs. (16) and (17) are slightly different, since computing the value of *α* that makes these contributions exactly equal is very difficult. However, the minor differences that our heuristic choice of *α* might introduce do not undermine the results of the experiments, since the approximate effect of modifying *α* is mildly shifting the curves in Figs. 4–6 to the left or right – which does not change the conclusions.

Our method also outperformed mutual information when applied to real data from the Allen Institute and BigBrain datasets, which are more challenging due to the more complex relationships between the two contrast mechanisms, and the presence of artefacts such as cracks and tears. Qualitatively speaking, the superiority of our approach is clearly apparent from Figs. 9 and 10, in which it produces much smoother segmentations and reconstructions in the orthogonal planes. We note that we did not introduce any smoothness constraints in the reconstruction, e.g., by forcing the registered histological sections to be similar to their neighbours, through an explicit term in the cost function of the registration. Such a strategy would produce smoother reconstructions, but these would not necessarily be more accurate – particularly if one considers that the 2D deformations fields of the different sections are independent *a priori*, which makes the histological sections conditionally independent *a posteriori*, given the MRI data and the image intensity transform. Moreover, explicitly enforcing such smoothness in the registration would preclude qualitative evaluation through visual inspection of the segmentation in the orthogonal orientations.

The proposed algorithm is hybrid in the sense that, despite being formulated in a generative framework, it replaces the likelihood term of the synthesis by a discriminative element. We emphasise that such a change still yields a valid objective function (Eq. (9)) that we can approximately optimise with VEM – which maximises Eqs. (10) and (11) instead. The VEM algorithm alternately optimises for *q* and *θ* in a coordinate descent scheme, and is in principle guaranteed to converge. In our method, we lose this property due to the approximate optimisation of the random forest parameters, since injecting randomness is one of the key elements of the success of random decision trees. However, in practice, our algorithm typically converges in 5–6 iterations, in terms of changes in the predicted synthetic image (i.e., in *μ_n**x**_* and $\sigma_{n\text{x}}^{2}).$

Our approach can also be used in an online manner, i.e., if data become progressively available at testing. For example, the random forest could be optimised on an (ideally) large set of images, considering them simultaneously in the framework. Then, when a new pair of images arrives, one can assume that the forest parameters are fixed and equal to $\hat{\theta }$, and proceed directly to the estimation of the synthetic image $\mu_{1\text{x},}\sigma_{1\text{x}}^{2}$ and deformation field ${\hat{\text{U}}}_{1\cdot }$ An alternative would be to fine tune *θ* to the new input, considering it in isolation or jointly with the other scans. But even if no other previous data are available (i.e., *N* = 1), the registration uncertainty encoded in *q* prevents the regression from overfitting, and enables our method to still outperform mutual information. This is in contrast with supervised synthesis algorithms, which cannot operate without training data.

The work presented in this paper also opens up a number of new directions of related research. One direction is integrating deep learning techniques into the framework, which could be particularly useful when large amounts of image pairs are available, e.g., in a large histology reconstruction project. The main challenges to tackle are overfitting and avoiding to make the algorithm impractically slow. A possible solution to this problem would be to use a pretrained network, and only update the connections in the last layer during the analysis of the image pair at hand (e.g., as in Wang et al., 2017). Another direction of future work is the extension of the algorithm to 3D. Albeit mathematically straightforward (no changes are required in the framework), such extension poses problems from the practical perspective, e.g., the memory requirements for storing *q* grow very quickly. Another avenue of future work is the application to other target modalities, such as optical coherence tomography (OCT).

Yet another interesting direction would be to explicitly model artefacts in the probabilistic model. While the method proposed here copes with cracks, holes, etc., by downweighting them in the registration, better results might be obtained by using more complex, non-diffeomorphic deformation fields which, combined with intensity models for missing tissue, could better represent these artefacts. In a similar fashion, a relevant direction of future work is the simulation of histological artefacts in images – possibly MRI slices, or histological sections with little or no artefacts. The existing literature on such simulations is surprisingly sparse, even though such synthetic images would enable us to quantitatively evaluate the performance of registration methods in presence of cracks, tears, folding, etc. Finally, we will also explore the possibility of synthesising histology from MRI. This a more challenging task that might require multiple input MRI contrasts, depending on the target stain to synthesise. However, synthetic histology would not only provide an estimate of the microanatomy of tissue imaged with MRI, but would also enable the symmetrisation of the framework presented in this article; by computing two syntheses, the robustness of the algorithm would be expected to increase.

The algorithm presented in this paper represents a significant step towards solving the problem of aligning histological images and MRI, by exploiting the connection between registration and synthesis within a novel probabilistic framework. We will use this method to produce increasingly precise histological reconstructions of tissue, which in turn will enable us to build probabilistic atlases of the human brain at a superior level of detail.

## Supplementary Material

Supplementary material associated with this article can be found, in the online version, at doi: 10.1016/j.media.2018.09.002.

Supplementary Material

## Figures and Tables

**Table 1:** List of parameters in model, values, and summary of criteria for setting them to their corresponding settings.

Symbol	Value	Description	Criteria for setting	Notes
*β*_1_	0.02	Weight of unary term in MRF	Visual inspection in pilot image	Equivalent to *σ* = 5 mm
*β*_2_	0.02	Weight of pairwise term in MRF	Heuristic: set *β*_2_ = *β*_1_	N/A
$\sigma_{k}^{2}$	0.5 mm	Variance of landmarks	Set to a low value	N/A
*a*	2	Shape parameter of Inv-Gamma	A couple of pseudo-observations	Equivalent to 4 pseudo-obs.
*b*	5^2^*a*	Scale parameter of Inv-Gamma	A small intensity sample variance	Equivalent to 4 pseudo-obs.
*T*	100	Number of trees in forest	More is better, but slower	N/A
N/A	5	Minimum samples in leaves	Most packages use 1–10	N/A
N/A	5	Features sampled at each node	Heuristic: sq. root of total features	N/A
*α*	2/(9|Ω_*n*_|)	Weight of proposed image term	Match range of mutual information	Cost = 1 if all pixels 3*σ* away
N/A	64	Bins for mutual information	NiftyReg default	N/A
*β_b_*	0.001	Weight of bending energy	NiftyReg default	Both for proposed and MI
*β_l_*	0.01	Weight of stretching / shearing	NiftyReg default	Both for proposed and MI
*β_j_*	0	Weight of Jacobian energy	NiftyReg default	Both for proposed and MI

**Table 2:** Mean, median and maximum registration errors on Allen dataset (in mm). The p-value corresponds to a paired, non-parametric, Wilcoxon signed-rank test comparing the landmark-wise errors produced by the two competing methods.

Method	Mean	Median	Maximum	p-value
Mutual info.	1.83	1.49	46.25	N/A
Proposed	1.49	1.22	18.45	4.4 · 10^−33^

**Table 3:** Mean, median and maximum registration errors on BigBrain dataset (in mm). The p-value corresponds to a paired, non-parametric, Wilcoxon signed-rank test comparing the landmark-wise errors produced by the two competing methods.

Method	Mean	Median	Maximum	p-value
Mutual info.	1.70	1.31	18.02	N/A
Proposed	1.41	1.19	14.09	5.4 · 10^−23^

## Footnotes

3 http://atlas.brain-map.org/atlas?atlas=265297126.
