# Supplementary material for: Joint registration and synthesis using a probabilistic model for alignment of MRI and histological sections
Source: Med Image Anal. Author manuscript; Available in PMC 2019 Sep 12. (PMC6742511; doi:10.1016/j.media.2018.09.002)

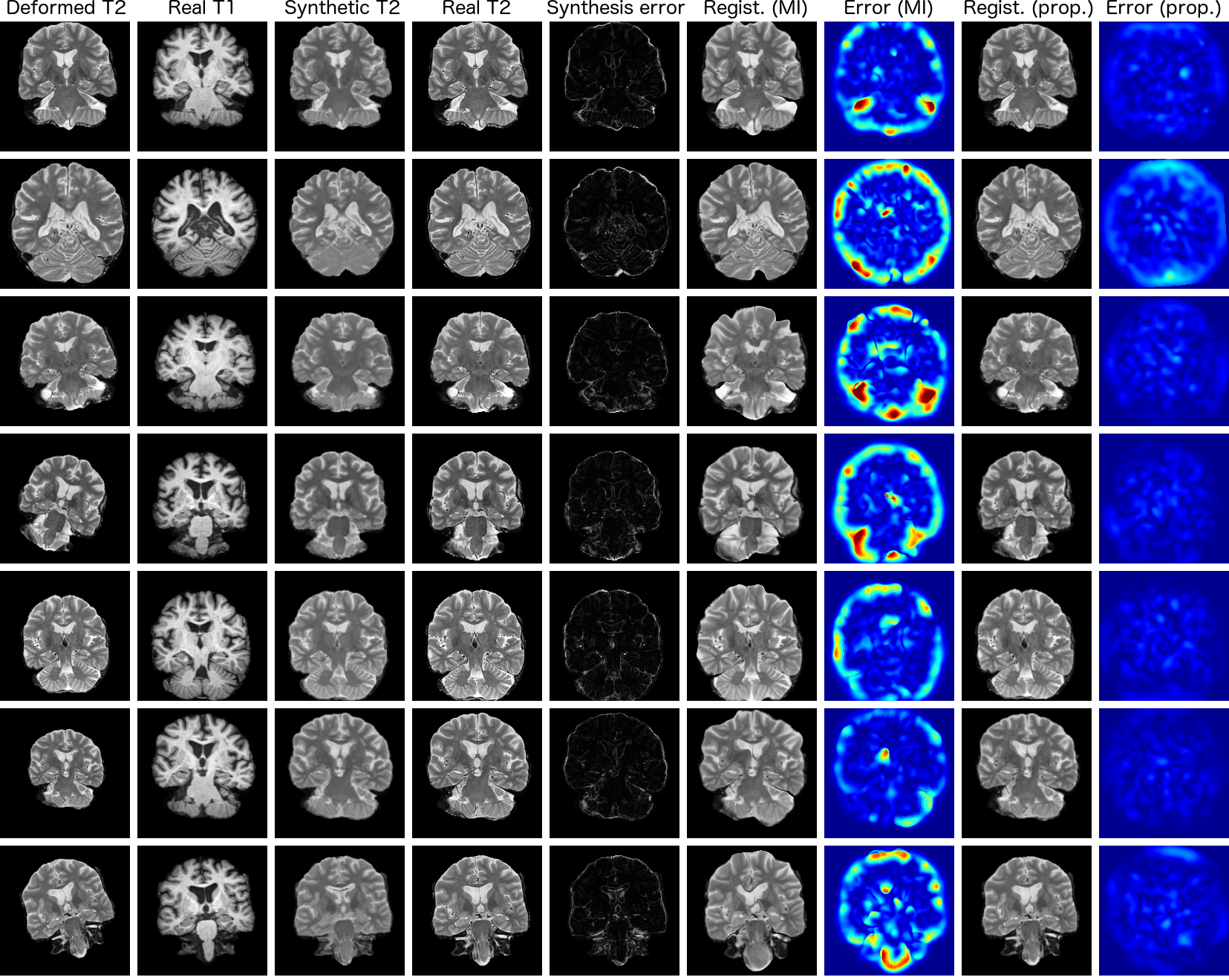


Figure S1: More examples from the synthetic dataset. Each row represents a different example. The columns display, from left to right: the randomly deformed T2 slice; the undistorted (“real”) T1; the synthetic T2 (mean $\mu_{nx}$); the real T2, for comparison; the synthesis error (i.e., absolute difference image); the registered T2 using mutual information alone; the corresponding error magnitude (i.e., magnitude of the difference between the synthetic and recovered deformation fields); the registered T2 using our proposed method; and the error magnitude with our approach. The control point spacing was 6 mm. In the registration error images, the color map spans from zero (dark blue) to 10 mm (or greater, in maroon).


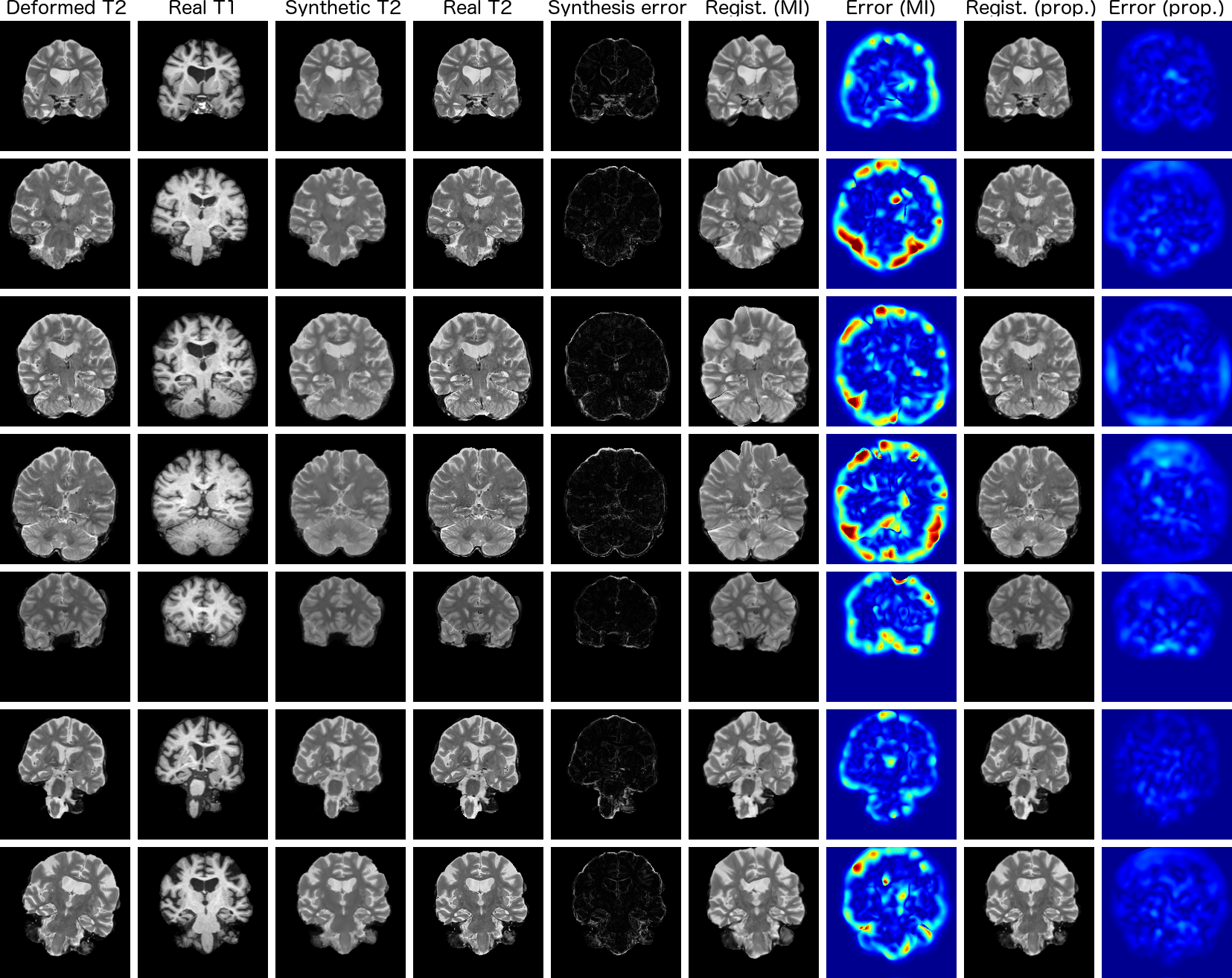


Figure S2: Additional examples from the synthetic dataset. Please see legend of Figure S1 for a description of this figure.

Figure S3: Sample orthogonal (sagittal) views of the reconstructed synthetic dataset. For each sample, we show (from left to right: original T1; original T2; distorted T2; recovered T2 with mutual information; and recovered T2 with our method. As in Figures S1 and S2, the control point spacing was 6 mm.

Figure S4: Additional examples from synthetic dataset (sagittal). Please see legend of Figure S3 for a description of this figure.

Figure S5: Sample orthogonal (axial) views of the reconstructed synthetic dataset. For each sample, we show (from top to bottom: original T1; original T2; distorted T2; recovered T2 with mutual information; and recovered T2 with our method. As in Figures S1-S4, the control point spacing was 6 mm.

Figure S6: Additional examples from synthetic dataset (axial). Please see legend of Figure S5 for a description of this figure.

*
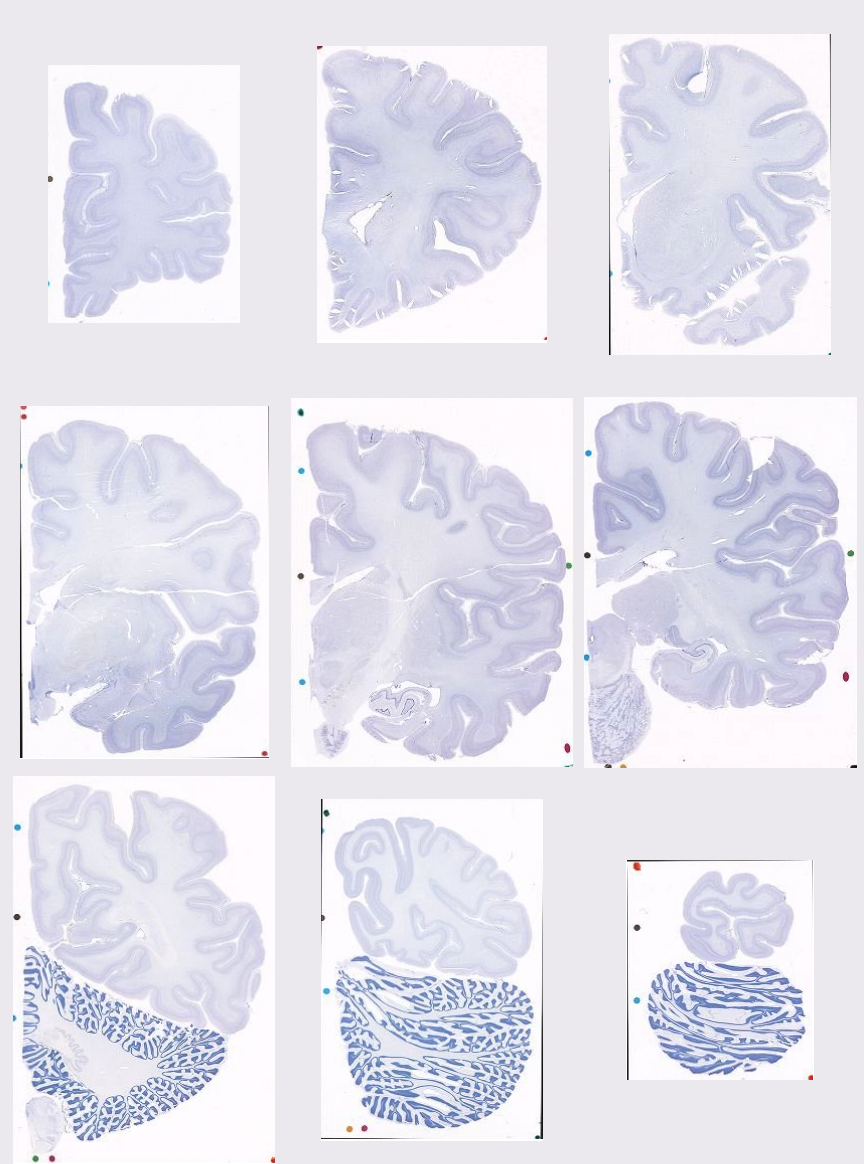
*

Figure S7: Histological sections from the Allen Institute atlas. The sections are coronal, and are sorted from anterior to posterior (left to right, top to bottom). These are some of the sections displaying the least artefacts – and yet exhibit noticeable cracks and small bit of missing tissue. Sections with more severe artefacts are show in Figure S8 below. The full dataset can be found at http://atlas.brain-map.org.

*
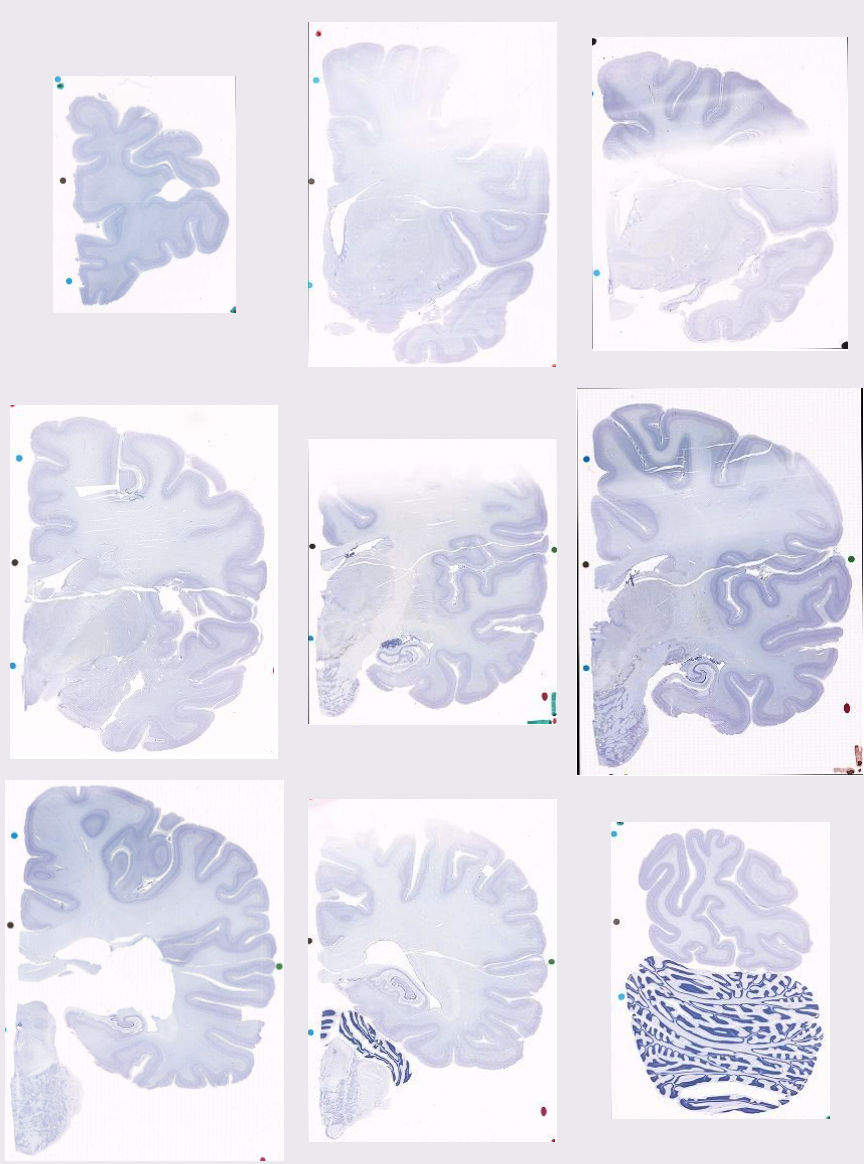
*

Figure S8: Additional sections from the Allen Institute atlas, displaying more sever artefacts – larger cracks, holes, uneven staining and missing tissue. The sections are coronal, and are sorted from anterior to posterior (left to right, top to bottom).

Figure S9: Histological sections from the BigBrain dataset, and corresponding (resampled) MRI slices. The sections are coronal, and are sorted from posterior to anterior (left to right, top to bottom). These are some of the sections displaying fewer artefacts. The full dataset can be found at http://bigbrain.loris.ca.

Figure S10: Additional sections from the BigBrain dataset (and corresponding MRI slices), displaying more sever artefacts, such as tearing and folding. The sections are coronal, and are sorted from posterior to anterior (left to right, top to bottom).

Equation S1: Objective function for variational E-step (Section 2.2.1).


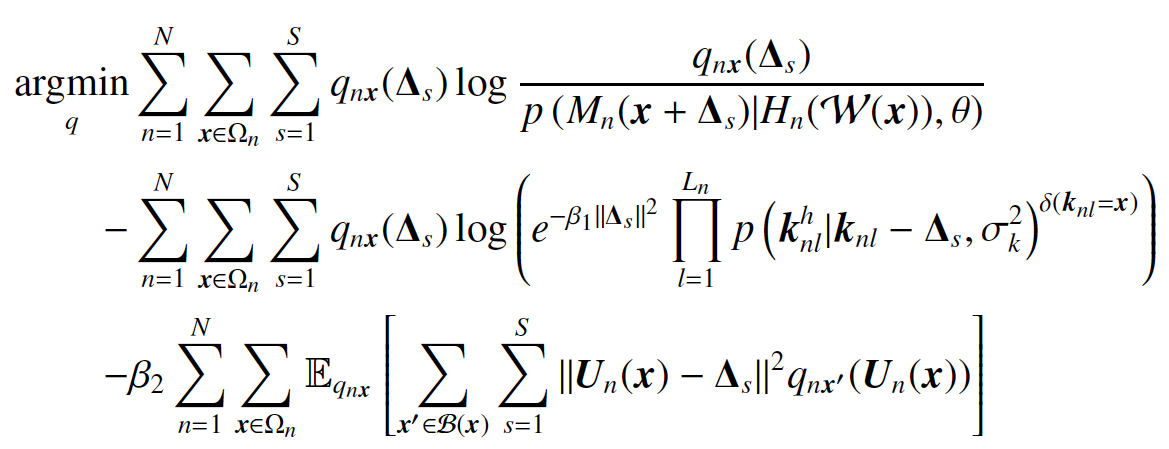


Equation S2: Objective function for computation of optimal deformation fields (section 2.2.2).


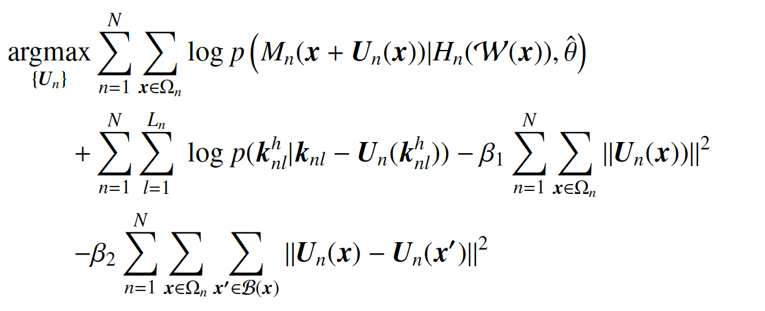

Supplement: Supplementary Material [file EMS84005-supplement-Supplementary_Material.docx]
